# Switching Between ILCT and ^3^MLCT Excited States by Complex Formation in Ruthenium–Polypyridine Complex Containing Thiacrown-Ether Unit

**DOI:** 10.3390/molecules31132213

**Published:** 2026-06-24

**Authors:** Sergey Tokarev, Anatoly Botezatu, Daria Kharkovskaya, Gediminas Jonusauskas, Yuri Fedorov, Olga Fedorova

**Affiliations:** 1 A.N. Nesmeyanov Institute of Organoelement Compounds, Russian Academy of Sciences, 119334 Moscow, Russia; botezatu@ineos.ac.ru (A.B.); daryakharkovskaya@yahoo.ca (D.K.); fedorov@ineos.ac.ru (Y.F.); fedorova@ineos.ac.ru (O.F.); 2 Laboratoire Ondes et Matière d’Aquitaine–UMR CNRS 5798, University of Bordeaux, 351 cours de la Libération, 33405 Talence, France; gediminas.jonusauskas@u-bordeaux.fr

**Keywords:** bimetallic complex, ruthenium(II) complex, complex formation, imidazophenanthroline, phosphorescence, MLCT

## Abstract

In this work, we report an example of tuning the photophysical properties of a polypyridine ruthenium(II) complex via the coordination of a second cation. A new ruthenium(II) complex contains a thiacrown-ether fragment that allows selective binding of additional metal cations (Ba^2+^, Cd^2+^, Pb^2+^), leading to pronounced changes in the optical and electronic properties of the bimetallic system. Spectroscopic and electrochemical studies reveal that the monoruthenium precursor displays dual excitation pathways involving either intraligand charge transfer (ILCT) or triplet metal-to-ligand charge transfer (^3^MLCT) excited states. Upon coordination of a second metal ion, the ILCT channel is suppressed, and only the ^3^MLCT state remains emissive, resulting in a significant increase in phosphorescence quantum yields (up to 22.6% in degassed solutions) for the bimetallic derivative. Time-resolved emission studies confirm the conversion from biexponential to monoexponential luminescence decay upon complexation. Electrochemical analysis and density functional theory (DFT) calculations support the hypothesis that cation binding alters the electron density distribution within the chromophore, stabilizing the MLCT pathway. These results demonstrate that incorporation of a second cation provides an effective strategy to control excited-state dynamics in ruthenium complexes, offering opportunities for the rational design of photosensitizers and photofunctional materials.

## 1. Introduction

Ruthenium–polypyridine complexes remain the target of significant research efforts as a result of their attractive photophysical and redox properties, which make them potentially useful as photosensitizers in photovoltaic devices [1], molecular wires [2], and sensors [3]. Their photosensitizing ability originates from the nature of the ruthenium(II) center, which enables the formation of a long-lived triplet metal-to-ligand charge transfer (^3^MLCT) excited state.

The ability of ruthenium to undergo reversible oxidation within coordination complexes has sparked considerable interest in designing bimodal supramolecular architectures that incorporate both photoinduced electron donor and acceptor units. In a work [4], one of the bipyridine ligands featured an extended π-conjugated framework of an acceptor nature, which enabled the formation of a more stable excited state. This structural design opened the possibility for storing photogenerated charges at more reducing potentials than the previous generations of Ru complexes. In other studies, the ruthenium bipyridine fragment was covalently linked to amino acid residues, which serve as intracomplex electron donors for the reduction in photo-generated Ru(III) [5,6].

Another obvious “partner” for ruthenium within a complex is a second metal cation. The reason is to design a molecular photocatalyst containing both a photoactive and a catalytic module. The bimetallic ruthenium(II)-manganese(II) complex was found to be one of the model systems of this type and a suitable starting point for the development of multinuclear RuMn_n_ complexes [7]. Another example would be a series of ruthenium-polypyridine complexes covalently bound to a terpyridine coordinating site for the Mn(II) ion [8]. Intramolecular electron transfer from Mn(II) to photogenerated Ru(III) was observed by time-resolved transient absorption and EPR. Trimetallic Ru_2_Rh and Ru_2_Ir complexes have also been investigated as photocatalysts, where Ru(II) acts as a photoinduced electron donor, while Rh(III) and Ir(III) serve as electron reservoirs for catalysis [8,9,10,11].

In a number of works by research teams [12,13,14], the photoelectron transferred from the excited state of the Ru moiety to the paramagnetic Cu^2+^ ion was reported when one of the ligands of the polypyridine Ru(II) complex interacts with copper(II). The photoelectron transfer (PET) leads to a decrease in the luminescence quantum yield of the ruthenium–polypyridine complex.

In some cases, the introduction of a second metal ion did not lead to the formation of PET but did affect the physicochemical properties of the ruthenium-polypyridine derivative. Thus, in [15], the authors attribute luminescence enhancement of the polypyridine Ru(II) complex upon binding of mercury(II) or silver(I) cations to an increase in the rigidity of the amide functionalized benzothiazole unit at the bipyridine framework as a result of coordination. As a consequence of inhibiting nonradiative decay, photoluminescence is enhanced. According to the authors, the luminescence enhancement of products is due to the population of emissive ^3^MLCT states upon excitation, while initial complexes undergo a transition to non-emissive metal-centered (^3^MC) states. Finally, computational work [16] predicts photoejection of an alkaline earth metal cation from the crown-ether fragment of the ligand of a bimetallic rhenium complex.

Thus, the introduction of a covalently bound organic fragment or a second metal cation significantly alters the photophysical characteristics and structure of ruthenium(II) complexes, both in strength and direction, which requires further study and systematization.

In previously published papers of our group, bimetallic ruthenium complexes of a new type with crown-containing imidazophenanthroline derivatives as a common ligand for two cations were reported. The paper [17] describes the application of such complexes to improve the performance of gas semiconductor sensors. It was shown that both cations in the composition of the bimetallic complex simultaneously manifest properties that improve the analysis process. Another paper [18] describes the synthesis and basic physicochemical characteristics of one such complex. It was shown that the introduction of Cu(II) cation into the Ru(II) complex with azadithia-15-crown-5 ether containing imidazo [4,5-f][1,10]phenanthroline weakly affects the electrochemical characteristics of the Ru(II)-containing part of the bimetallic complex and slightly changes the absorption spectrum of the monometallic complex, but substantially increases the luminescence intensity.

**Scheme 1:**
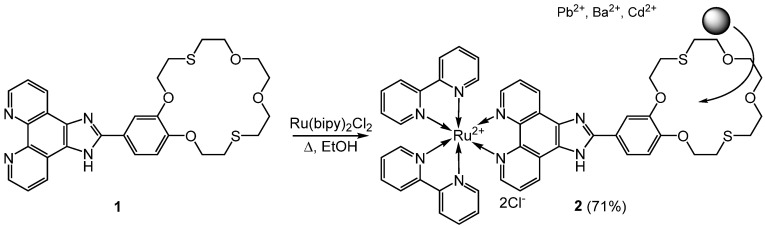
Formation of bimetallic complexes.

Herein, we report on the preparation and studies of heterobimetallic complexes with a series of metal cations based on the monoruthenium complex **2** (Scheme 1). The chosen ligand **1** contains different coordination centers: 1,10-phenanthroline moiety and dithia-18-crown-6 ether fragment. The ruthenium(II) will occupy the N,N-moiety and the dithia-18-crown-6 will remain unoccupied to bind the second cation. Coordination sites are distant from each other to prevent electrostatic repulsion of cations, but are linked into one conjugated system so that the coordination of the second cation with the crown ether fragment should affect the electron distribution of the chromophore system of the initial monoruthenium complex. Dithia-18-crown-6 ether fragment contains both soft (S-atom) and hard (O-atom) Lewis bases, so it should coordinate different types of cations. We chose 3 different cations to test all types of Lewis acids (Ba^2+^, Cd^2+^, Pb^2+^) upon coordination, depending on differences within the cations. The selected cations also vary in size from the smallest Cd(II) to the largest Ba(II) [19].

The synthesis of ligand **1**, ruthenium complex **2** and some of its bimetallic derivatives is described in [17]. However, physicochemical properties of the ligand, mono- and bimetallic complexes have been given little to no attention.

## 2. Results

### 2.1. Basic Physicochemical Properties

**Figure 1:**
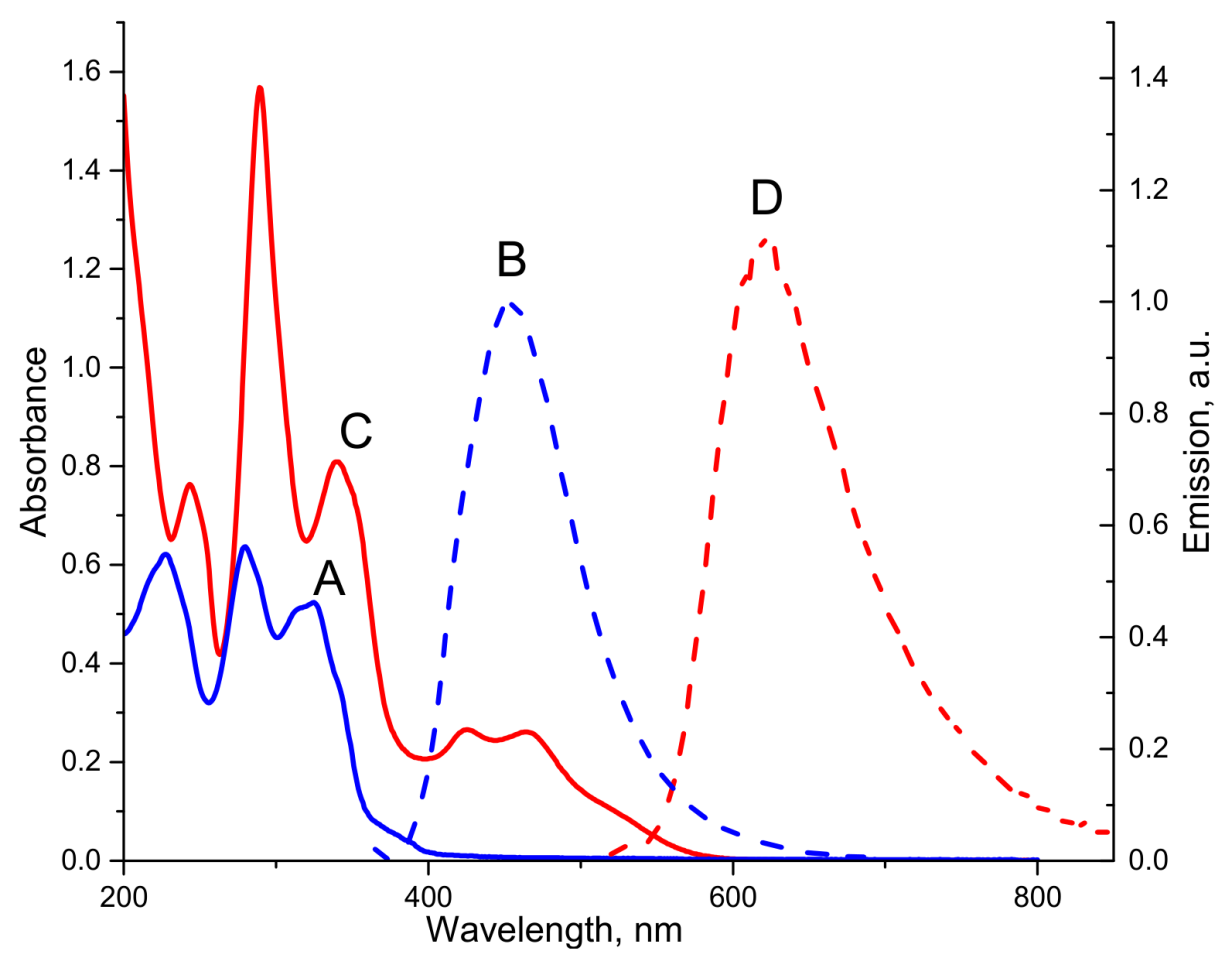
Absorption (solid lines) and emission (dotted lines) spectra of **1** (A and B correspondingly), **2** (C and D correspondingly) in acetonitrile solutions. C**_1_**_or **2**_ = 2 × 10^−5^ mol·L^−1^.

The absorption and emission spectra of **1** and **2** are shown in Figure 1. Ligand **1** shows several strong absorption bands in the region shorter than 400 nm. For complex **2,** the absorption peaks in the UV region correspond to ligand-centered π-π* transitions. They roughly match with transitions of free ligand **1**. π-π* Transitions of 2,2’-bipyridine (bipy) ligands and ligand **1** are superimposed at the band near 290 nm. [20] Long-wavelength intraligand transition of imidazophenanthroline (ImPh) ligand is represented in a broad band in the 320–390 nm region. In the visible region, complex **2** displays a broad absorption band of complex structure, which should be assigned to metal-to-ligand charge transfer (^1^MLCT) transitions.

Both ligand **1** and complex **2** exhibit broad emission with maxima around 450 and 620 nm, respectively. At room temperature in air-saturated acetonitrile solution, the luminescence quantum yield of complex **2** is rather low, probably due to the fact that emission often occurs from the ^3^MLCT state for such complexes. [21] The triplet excited state of Ru(II) complexes transfers energy to the dissolved oxygen molecules, which leads to emission quenching [22]. Quantum yield of **2** in the degassed solution at room temperature increases by 7 times (Figure S15). Optical properties of **1** and **2** are listed in Table 1.

Cyclic voltammograms of **1** and **2** are shown in Supplementary Materials (Figures S1 and S2). Electrochemical properties of **1** and **2** are listed in Table 1. The oxidation of **1** proceeds via several steps, with the initial steps presumably including sulfur atoms of the crown-ether fragment, since it was shown that heteroatoms of crown-ether are the most easily oxidized sites for a similar ligand with azadithia-15-crown-5 ether moiety [23].

Voltammogram of ruthenium(II) complex **2** demonstrates four oxidation waves and four reduction waves. Low irreversible oxidation waves in the 0.76–1.10 V range should be attributed to the overlay of **1** and chloride counterions oxidations [24]. The last quasireversible oxidation at 1.4 V matches with metal-centered oxidation reported for [Ru(bipy)_3_]^2+^ [25] and other Ru(II) polypyridil complexes [26]. Thereby, it was also attributed to the one-electron oxidation of the ruthenium(II) cation. All of the reductions are ligand-centered and reversible except for the last one at −2.42 V vs. Ag/AgCl reference electrode. The first three reversible reduction waves were attributed to bipy ligands, since recorded potentials are close to those reported for [Ru(bipy)_3_]^2+^. [25] The fourth reduction corresponds to the ImPh ligand, since its position and irreversibility are very close to the reduction recorded for **1**. We used the first reduction potential to estimate the LUMO energy.

### 2.2. Bimetallic Complex Formation Study

**Figure 2:**
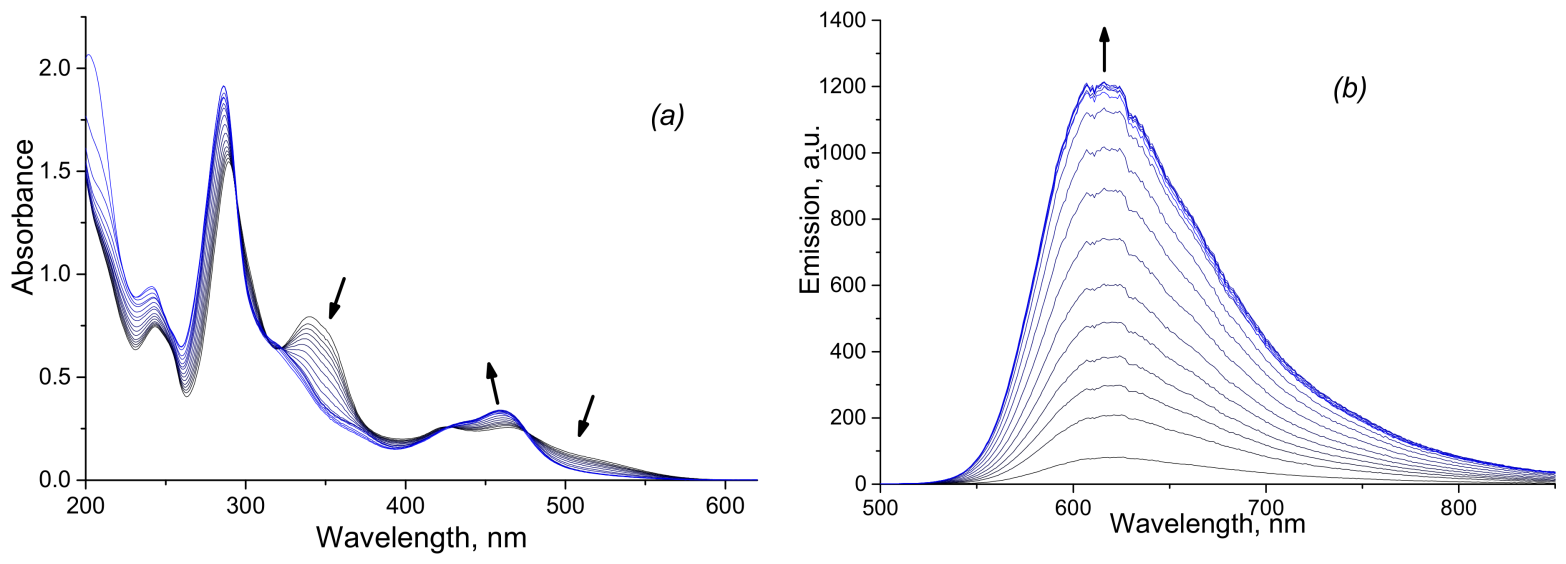
Electronic (**a**) absorption and (**b**) emission spectra of a MeCN solution of complex **2** at various concentrations of lead perchlorate. The initial concentration of **2** C_2_ = 2 × 10^–5^ mol·L^−1^, and the concentration of salt varies in the range of 0–4 × 10^−3^ mol·L^−1^. The arrows indicate change in the spectra with increasing concentration of lead perchlorate.

The addition of perchlorates of different metals causes significant changes in the optical properties of complex **2**. The successive addition of small portions of lead (II) perchlorate to the acetonitrile solution of **2** leads to noticeable changes in the absorption and especially in the luminescence spectra, which indicates complex formation. (Figure 2a) Upon complexation, the intraligand π-π* transition band of the ImPh ligand at 340 nm undergoes a hypsochromic shift and, as a result, overlaps with the intraligand π-π* band of bipy at 290 nm. The hypsochromic band shift is due to the fact that the interaction of the lead cation with heteroatoms of the crown ether fragment leads to a decrease in the electron-donating function of oxygen atoms directly included in the chromophore part of the molecule [27]. There were also some changes in the visible region, namely, the long-wavelength slope of the broad MLCT band narrowed, and the absorption maxima at 460 nm increased slightly. The position of the maximum and the shape of the luminescence spectrum remained unchanged upon complexation of **2** with Pb^2+^, although the quantum yield in an air-saturated solution increased from 0.5% to 3.5%.

In case of titrations with Cd^2+^ and Ba^2+^ (Figures S3 and S4), changes in both the visible and near UV regions are of the same type: a small increase in absorption of the long-wavelength MLCT band, reduction in the broad longwavelength slope, change in the π-π* transition band within the ImPh ligand and noticeable increase in emission intensity with preservation of the shape and maximum of the spectrum. Of particular interest is that the nature of the second metal cation does not affect the optical changes in the absorption and luminescence spectra observed during complexation.

Calculated stability constants of bimetallic complexes are presented in Table 2. It is noteworthy that the nature of the metal cation has some effect on the binding strength, but less than would be expected. For example, the stability constants of complex **2** with both barium(II) (hard Lewis acid) and cadmium(II) (soft Lewis acid) are very similar. The most stable complex was formed with Pb^2+^, probably due to its intermediate nature.

Additionally, complex formation was studied by NMR spectroscopy and cyclic voltammetry (data are presented in Supplementary Materials, Figures S5–S11). An EXAFS experiment was also performed for solutions of the complexes and elemental analysis of precipitates obtained by mixing solutions of **2** and perchlorates of lead(II), barium(II) and cadmium(II) (Supplementary Materials, Figures S12–S14 and Table S2) to prove the strong coordination of the second cation both in solution and in the solid phase.

**Figure 3:**
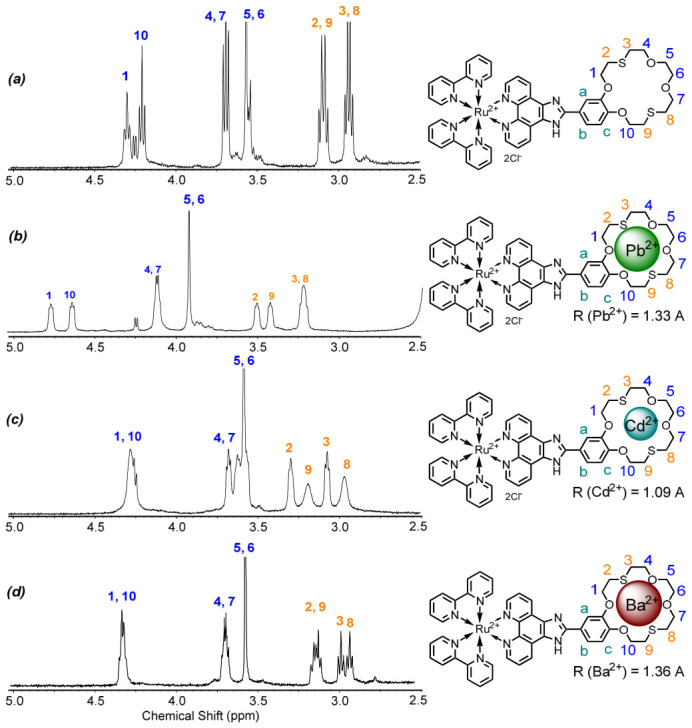
^1^H NMR spectral changes in crown-ether signals of complex **2** (**a**) upon the addition of 1 equivalent of lead(II) perchlorate (**b**), cadmium(II) perchlorate (**c**) or barium(II) perchlorate (**d**) in d-MeCN.

In ^1^H NMR spectra, signals of all crown-ether protons of **2** uniformly moved downfield upon the addition of one equivalent of lead perchlorate (Figure 3b). In the case of the addition of cadmium perchlorate to complex **2**, a downfield shift was also observed, but noticeably weaker than for lead (Figure 3c). The largest changes were observed for the proton signals of CH_2_ groups near sulfur atoms. Barium causes even weaker changes (Figure 3d). In this case, the largest shift is for protons 1,10 near the oxygen atoms. Nevertheless, comparison of the NMR spectra of monoruthenium complex **2** and its derivative bimetallic complexes proves that the coordination of the second cation occurs upon the crown ether fragment of the molecule. From NMR data, we should also point out that only one coordination product of high stability forms in each case, since all NMR spectra of bimetallic complexes contain only one set of signals that differs from the spectrum of **2**. Full NMR spectra of **2** and its bimetallic derivatives are presented in Supplementary Materials Figures S9–S11.

### 2.3. Photophysical and Electrochemical Study of Bimetallic Complexes

**Figure 4:**
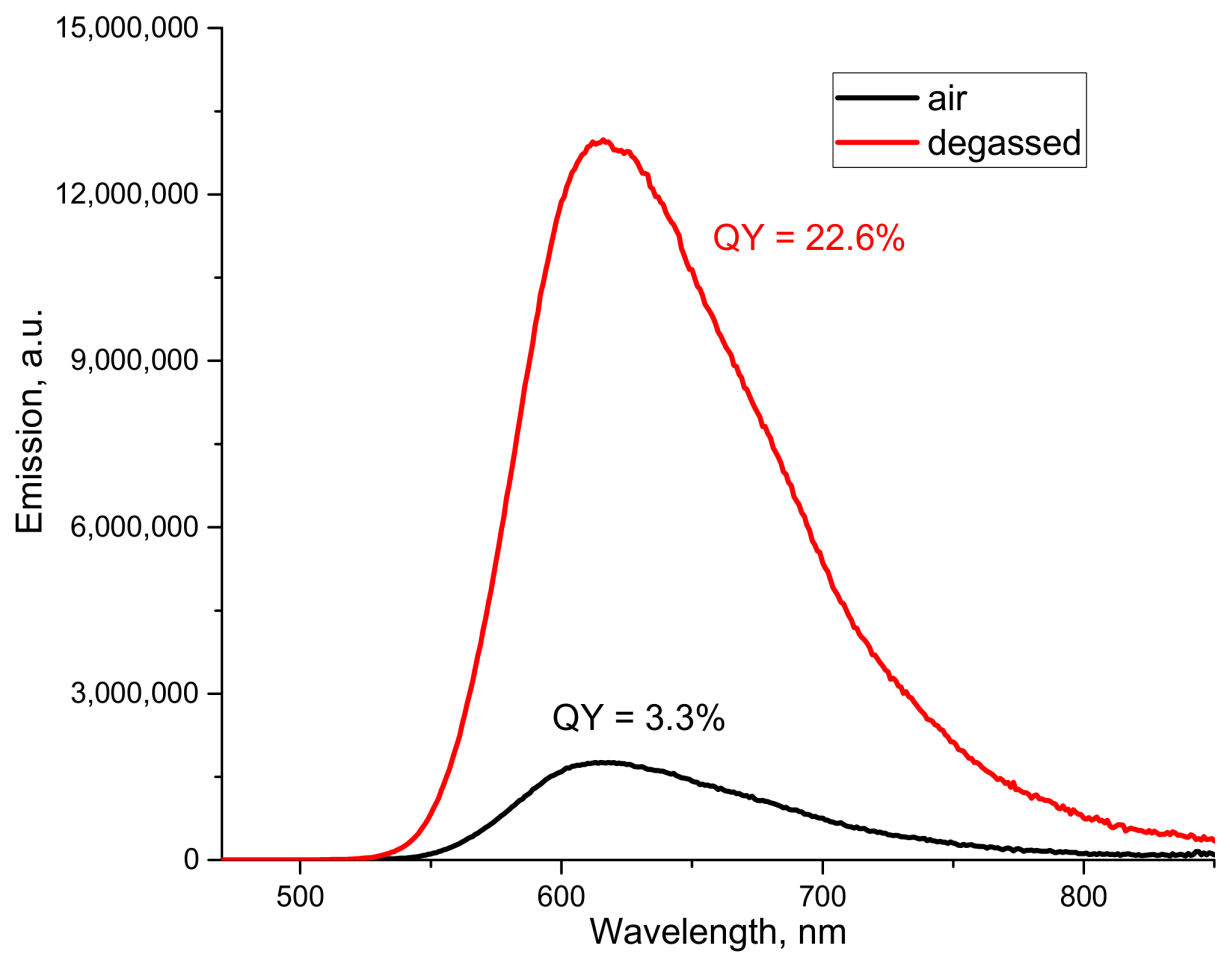
Emission spectra of a mixture of complex **2** with a 2-fold excess of Pb(ClO_4_)_2_ in air-saturated (black) and degassed (red) MeCN solution. Excitation at 460 nm.

The emission spectra of bimetallic complexes of ligand **2** with Pb^2+^, Ba^2+^ and Cd^2+^ ions are characterized by a wide band with maxima in the region of 615–620 nm, which coincides with the position of the luminescence band of the original monoruthenium ligand **2** (Figure 1 and Figure 4 and Figures S3 and S4 in Supplementary Materials). Degassing solutions of bimetallic complexes increases their quantum yields by 6–10 times (3.5 → 21.0%, 3.3 → 22.6%, 2.1 → 22.0% for **2**∙Pb^2+^, **2**∙Ba^2+^_,_ **2**∙Cd^2+^ correspondingly), but does not affect the shape and position of the maximum of the luminescence spectrum (Figure 4 and Figures S16–S18 in Supplementary Materials). A significant increase in luminescence intensity upon transition from air-saturated to degassed solutions of bimetallic complexes allows for the assignment of this emission to phosphorescence. It should be noted that for all bimetallic complexes, the luminescence quantum yields are several times higher than for the monoruthenium complex **2** in both air-saturated and degassed solutions. Thus, the transition from monoruthenium complex **2** to bimetallic complexes is accompanied by a drastic increase in the quantum yield of luminescence.

**Figure 5:**
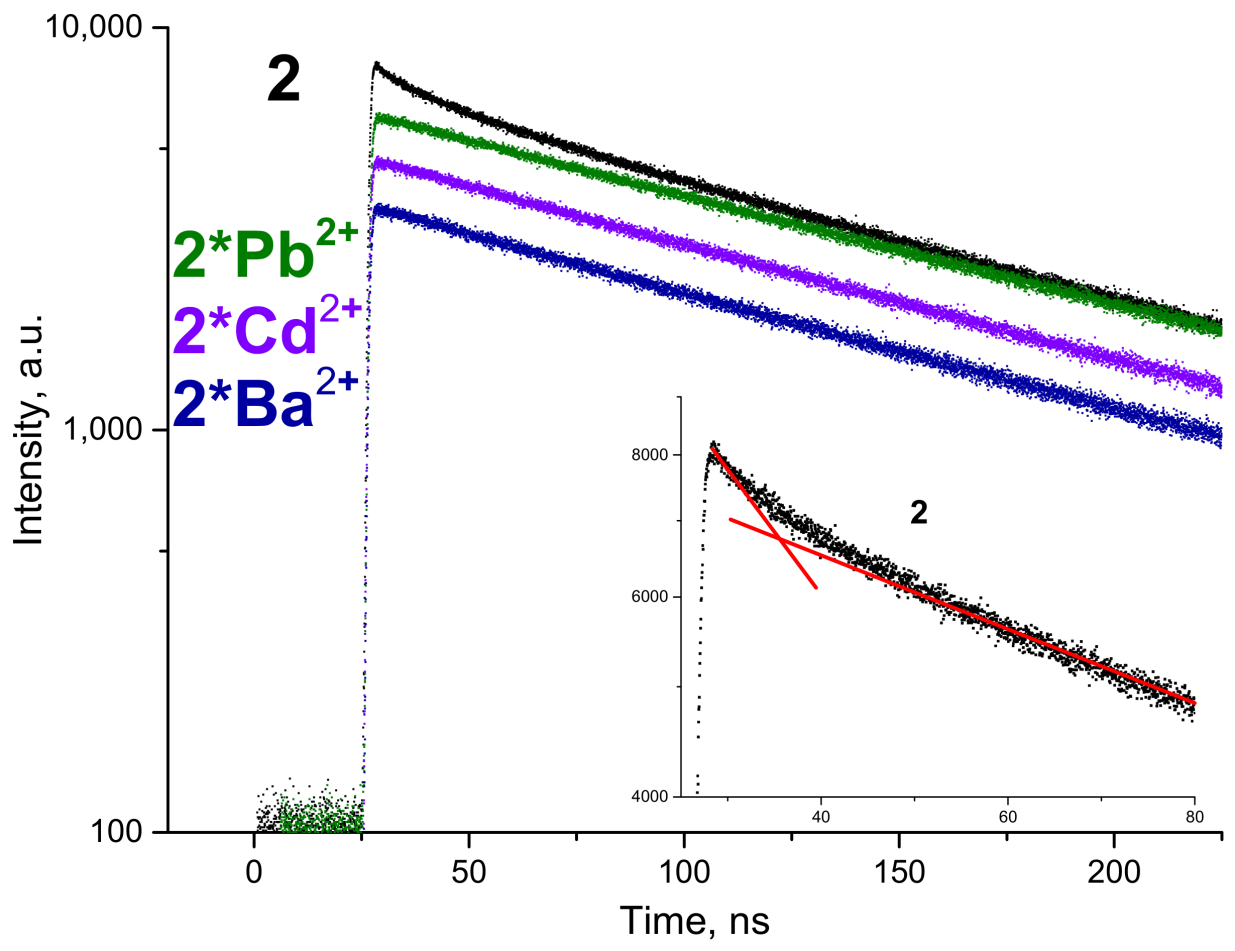
Time-resolved emission decay of **2**, **2**∙Pb^2+^, **2**∙Ba^2+^ and **2**∙Cd^2+^ (λ_exc_ = 460 nm, λ_em_ = 620 nm) in acetonitrile in air. Inset: time-resolved emission decay of the same solution of **2** on the time scale 25–80 ns. Red lines represent two components of the emission of **2**. The asterisk symbol in the figure denotes the complexation of **2** with a metal cation.

We have studied the emission decay of **1**, **2** and their bimetallic derivatives on a scale of 0–250 ns both in air-saturated and degassed acetonitrile solutions. The calculated lifetimes are presented in Table 3 and fitted curves for all complexes in Figures S19–S27 in Supplementary Materials. Figure 5 shows the emission decay curves for **2** and its bimetallic complexes in air-saturated solutions. The monoruthenium complex **2** exhibits dual-component emission with lifetimes differing by an order of magnitude. On the decay curve, this appears as a combination of two straight lines in half-logarithmic coordinates (marked by the red lines on the inset of Figure 5). In the degassed solution, the phosphorescence of complex **2** from ^3^MLCT is greatly enhanced, and its lifetime is further increased. Nevertheless, it is still possible to detect the short component, albeit with poorer accuracy. Under the same conditions, all bimetallic derivatives of **2** show only monoexponential decay with phosphorescent lifetimes.

**Scheme 2:**
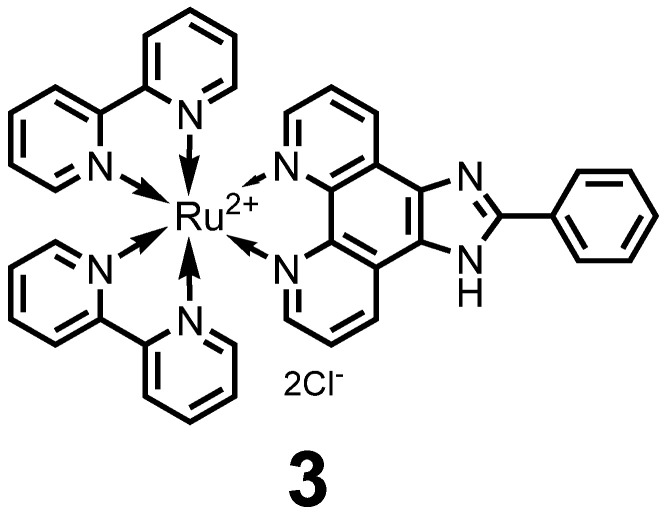
Structure of complex **3** without crown-ether.

**Scheme 3:**
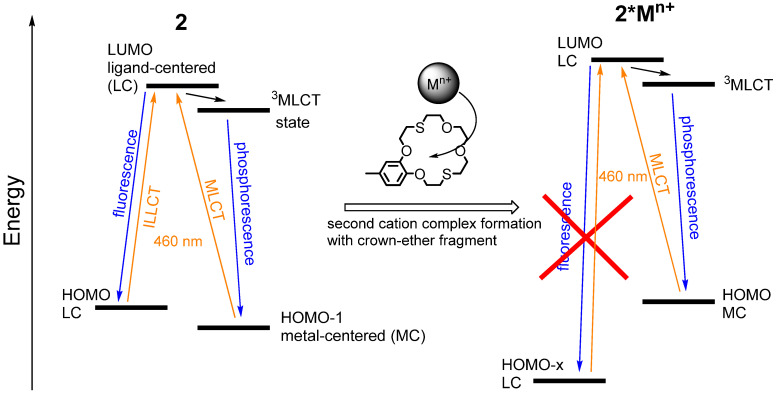
Molecular orbitals diagram of complex 2 and its bimetallic derivatives. The asterisk symbol in the figure denotes the complexation of **2** with a metal cation.

To explain the luminescent behavior of **2** and its bimetallic derivatives, we proposed the following hypothesis: irradiation of the initial monoruthenium(II) complex **2** with 460 nm leads to two possible processes: one that results in a phosphorescent ^3^MLCT excited state and the other of intra- or interligand nature that leads to a non- or very weakly emitting state. These transitions are competitors for excitation energy, and the presence of the latter reduces the probability of phosphorescence and hence the quantum yield. Coordination of the second cation on the crown ether moiety “anchors” the electron density of the ImPh ligand and lowers the energy of the highest ligand-centered molecular orbital. As a conclusion, the HOMO is now probably located on ruthenium, and the lowest transition producing steady state absorption corresponds to MLCT from ruthenium(II) towards bipy. Correspondingly, the excitation at 460 nm produces only ^1^MLCT excited state relaxing in a few tens of femtoseconds to ^3^MLCT*; this latter state relaxes to the ground state with emission of phosphorescence. The overall quantum yield rises since there is no competitor to draw excitation energy from phosphorescence. Binding of the second cation by the crown-ether moiety “transforms” complex **2** in a photophysical sense into complex **3** (Scheme 2), which contains no donor alkoxy groups. Complex **3** has a higher quantum yield than **2**, as well as an exclusively triplet excited state, according to time-resolved luminescence studies [28]. Our hypothesis is summarized in Scheme 3.

So, based on the calculated lifetimes and the proposed hypothesis, we attribute the short luminescent component for complex **2** to emission from 1ILLCT, and the long component belongs to the emission from ^3^MLCT. It is important to note that the short component is not the fluorescence of the ImPh ligand. The lifetime for ligand **1** was measured separately with great precision, and it does not match the short component for **2**. Thus, the short component originates from ILLCT within the complex. Bimetallic complexes, both in air and in degassed solutions, demonstrate monoexponential decays with lifetimes corresponding to emission from ^3^MLCT [28].

**Figure 6:**
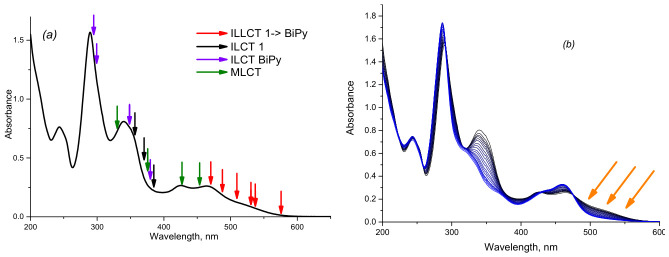
(**a**) Steady-state absorption spectrum of complex **2**, indicating the nature and approximate position of the absorption bands obtained from the CVA; (**b**) absorption spectrum of a MeCN solution of complex **2** at various concentrations of barium(II) perchlorate; orange arrows indicate the disappearance of the ILLCT absorption bands upon barium complexation.

To prove the hypothesis, we compared electrochemical data with steady-state absorption. A detailed analysis is provided in the Supplementary Materials, in the section “Comparison of electrochemical data with absorption spectroscopy results.” Figure 6a presents the absorption spectrum of complex **2**, with tentative assignments and positions of the absorption bands based on the combined data. In the 460 nm region, both ILLCT and MLCT bands overlap. Figure 6b shows the titration of complex **2** with barium(II) cation, leading to the formation of a bimetallic complex. Notably, the long-wavelength ILLCT bands disappear (shown with orange arrows), and only MLCT absorption remains in the 460 nm region.

Finally, the structure and boundary molecular orbitals (MO) were calculated by means of DFT and the Gaussian program version 9.5. Localization of 4 boundary MOs for **2** and **2**∙Pb^2+^ is shown in Table 4, and for **2**∙Ba^2+^, **2**∙Cd^2+^ at Table S3 in Supplementary Materials since they are similar to **2**∙Pb^2+^. According to the calculations, for monoruthenium complex **2,** the HOMO → LUMO transition is of ILCT and ILLCT nature, which does not lead to the formation of a ^3^MLCT state, which is usually responsible in such complexes for phosphorescence. It is possible that such an excited state relaxes via fluorescence. The HOMO-1 → LUMO transition, in turn, has a classical MLCT nature and is responsible for the observed phosphorescence. For the bimetallic complexes, the localization of HOMO changes from ligand **1** to the ruthenium(II) cation, while LUMO still remains distributed between the phenanthroline fragment of **1** and bipy. As a result, the HOMO → LUMO transition excites the ^3^MLCT state, and there are no other types of transitions between the boundary orbitals.

Thus, the calculation results support the hypothesis of the formation of two types of excited states for monometallic **2** under 460 nm excitation of MLCT and ILLCT nature. As for bimetallic derivatives, only MLCT has emerged.

To gain insight into the nature of the electronic transitions, the absorption spectra of **2** and **2**∙Pb were simulated in acetonitrile using TD-DFT based on optimized ground-state geometries. The computed spectra agree well with the experimental data (Figures S30 and S31), and the selected excitation energies, oscillator strengths, and dominant excitation character are compiled in Table S4.

To further characterize the excited states, natural transition orbital analysis was performed based on the calculated transition density matrices [29]. This approach provides the most compact description of the density rearrangement that occurs upon excitation, representing each transition as a single hole–electron pair. As anticipated, the lowest-lying excitations in both complexes exhibit a predominantly MLCT character. Only for **2** do the S_1_ and S_2_ states display a minor contribution from a ligand-to-ligand charge transfer transition, specifically from π_(ImPh)_ to π*_(bPy)_.

Notably, the MLCT energies remain largely unchanged when the heterobimetallic adduct forms. This invariance is consistent with the observation that the main contributor to the absorption band between 420 and 500 nm is the d_(Ru)_ → π_(bPy)_ transition, rather than the d_(Ru)_ → π_(ImPh)_ excitation (Table 5, first two lines). In contrast, the most pronounced changes in the excited-state character are observed for the ligand-centered transitions. For instance, for **2,** the bands near 350 nm appear from the intraligand charge transfer transition undergo a blue shift of approximately 20 nm upon formation of **2**•Pb, and its character evolves from a pure ILCT to a mixed ILCT/LLCT) excitation (Table 5, second two lines). It is noteworthy, however, that the formation of the bimetallic complex has a negligible effect on the oscillator strengths of these transitions. These computationally predicted changes in the ligand-centered transitions correlate well with the experimental UV-Vis spectra, where a comparable blue shift is observed in the same region, as well as with the CV data, which show a corresponding modulation of the ligand-based redox potentials upon coordination of a second cation via a crown-ether fragment.

## 3. Materials and Methods

Reagents and solvents were purchased from Sigma-Aldrich (Munich, Germany), Fluka (Seelze, Germany) and Reakhim (Moscow, Russia) companies. Most of them were used without further purification, except the NBu_4_PF_6_ (TBAHFP) for electrochemical analysis, which was used as a supporting electrolyte after recrystallization from wet ethanol and rigorous drying. Anhydrous acetonitrile (MeCN) for electrochemical and optical measurements was of spectroscopic grade purity.

NMR spectra were recorded on “INOVA-400” (Varian, Palo Alto, CA, USA) and Bruker Avance 400 (Karlsruhe, Germany) spectrometers (400.13 and 100.13 MHz frequency for 1 H and 13C, respectively). Chemical shifts are given with respect to the residual proton signal of the used solvent, i.e., 1.94 ppm for CD_3_CN, 2.49 ppm for DMSO-d6, 3.31 ppm for methanol-d4; ^13^C: 1.32 ppm for CD_3_CN, 39.52 ppm for DMSO-d6, 49.00 ppm for methanol-d4.

ESI mass spectra (ESI-MS) were acquired on a Finnigan LCQ Advantage tandem dynamic mass spectrometer (San Jose, CA, USA) equipped with a mass analyzer with an octapole ionic trap, an MS Surveyor pump, a Surveyor autosampler, a Schmidlin-Lab nitrogen generator (Geneva, Switzerland), and a system of data collection and processing using the X Calibur program, version 1.3 (Finnigan). The mass spectra were measured in the positive ion mode. Samples in MeCN were injected directly into the source at a flow rate of 50 µL min^−1^ through a Reodyne injector with a loop of 20 µL. The temperature of the transfer capillary was 150 °C, and the electrospray needle was held at a potential of 4.0 kV.

UV-Vis spectra were recorded on a Cary 300 spectrometer (Santa Clara, CA, USA), and for the fluorescence spectra, a Cary Eclipse spectrofluorometer (Santa Clara, CA, USA) was used. UV-Vis and fluorometric titration have been performed as follows: to 2.5 mL of the 2 × 10^–5^ M solution of a ruthenium complex **3** or **4** portions of 10^–3^ M of Ag(ClO_4_), Cd(ClO_4_)_2_, Cu(ClO_4_)_2_ or Pb(ClO_4_)_2_ solutions have been added successively to maintain a noticeable changes in a spectra of the resulting solution; the titration ended when there was no noticeable change in the spectrum upon the addition of the portion of the titration solution. Sets of the UV-Vis spectra have been processed by the SPECFIT 32 software version 3.0.37. All measured luminescence spectra were corrected for nonuniformity of detector spectral sensitivity. Rhodamine 101 (φ_fl_ = 1) and quinine bisulfate in 1N H_2_SO_4_ were used as references for the luminescence quantum yield measurements. The luminescence quantum yields were calculated using the equation:

$$\varphi_{i}=\varphi_{0}\frac{(1-10^{-A_{0}})*S_{i}*{n_{i}}^{2}}{(1-10^{-A_{i}})*S_{0}*{n_{0}}^{2}},$$

where φ_i_ and φ_0_ are the luminescence quantum yields of the studied solution and the standard compound, respectively; A_i_ and A_0_ are the absorptions of the studied solution and the standard, respectively; S_i_ and S_0_ are the areas underneath the curves of the luminescence spectra of the studied solution and the standard, respectively; and n_i_ and n_0_ are the refractive indices of the solvents for the substance under study and the standard compound (n_i_ = 1.3288, acetonitrile; n_0_ = 1.361, ethanol; n_i_ = 1.3391, 1N H_2_SO_4_).

Electrochemical measurements were carried out at 22 °C with a Metrohm Autolab B.V. potentiostat (Utrecht, The Netherlands). Cyclic voltammetry experiments were performed in a three-electrode cell equipped with a glassy carbon (GC) electrode (disk d = 2 mm), Ag/AgCl/KCl (aq. saturated; reference electrode), and a platinum electrode (counter electrode). Compounds were dissolved in degassed dry CH_3_CN containing TBAHFP as the supporting electrolyte (0.1 M). Dry argon gas was bubbled through the solutions for 30 min before cyclic voltammetry experiments. The scan rate was 200 mV s^−1^.

Flash column chromatography was carried out on a Biotage Isolera Prime chromatograph (Uppsala, Sweden). Aluminum oxide was hand-packed into the cartridge using the wet method.

Elemental analysis was performed in the A.N. Nesmeyanov Institute of Organoelement Compounds of the Russian Academy of Sciences.

## 4. Conclusions

The photophysical behavior of a new type of bimetallic ruthenium-containing complex based on crown- and phenanthroline-containing ligands has been studied in this work. In the examined complexes, ruthenium(II) is coordinated by the 1,10-phenanthroline moiety and dictates the optical behavior of the complex. The second cation is introduced into the macrocyclic cavity in such a way that its presence significantly increases the phosphorescence and quantum yield of the ruthenium(II) bimetallic complex, rising up to 21.0%, 22.6%, and 22.0% for **2**∙Pb^2+^, **2**∙Ba^2+^, and **2**∙Cd^2+^ correspondingly in degassed solution. It was shown that for the monoruthenium complex **2,** long-wavelength excitation leads to two different excitation pathways. Only one of which results in the ^3^MLCT* excited state, responsible for phosphorescence. The second excited state of ^1^ILLCT nature “takes away” the excitation energy from the emitting ^3^MLCT state, lowering the quantum yield. The introduction of a positively charged second cation into the crown ether significantly affects the electron density distribution of the boundary orbitals, making electron transfer from the ImPh ligand within the complex much more difficult. As a result, long-wave irradiation induces only the MLCT transition, which markedly increases the probability of relaxation via photon emission. In the case of the studied series of different metal ions (Ba^+2^, Pb^2+^, Cd^2+^), the observed effect of luminescence enhancement is not cation-selective. It could be explained by the fact that only oxygen atoms of the thiacrown ether unit are involved in the conjugation with the phenanthroline-containing ligand; the interaction of metal ions with these heteroatoms mainly affects the electron distribution in the Ru-complex. According to the NMR insight, all studied metals coordinate with oxygen heteroatoms, which causes the shifts in protons of methylene groups connected with oxygen (Figure 3). Also, it is important that the interaction of Ba^2+^, Pb^2+^, Cd^2+^ with the thiacrown ether fragment of the Ru(II) complex occurs with closed stability constants. The present work is focused on the photophysical and electronic effects accompanying the formation of bimetallic complexes. Aspects related to recyclability, long-term stability, reversibility, detailed selectivity screening, and practical concentration limits—while important for potential sensing applications—are beyond the scope of this study and will be addressed in subsequent work. In addition, DFT calculations showed a similar phenomenon for all three studied bimetallic complexes, that upon binding to second metal ions, the conversion from the ILCT excited state to the highly emissive ^3^MLCT triplet state occurs. The conversion from ILCT to LLCT/MLCT excited state by heavy metal ion binding in Iridium(III) complexes has been described earlier [30]. For the polypyridine complexes of Ru(II), this phenomenon has not been observed earlier.

## Figures and Tables

**Table 1:** Optical and electrochemical properties of **1** and **2**.

Comp.	λ_abs_, nm	λ_em_, nm	Φ, % ^a^	E_ox_ ^b^, V	E_red_ ^b^, V	HOMO ^c^, eV	LUMO ^c^, eV	ΔE_g_ ^c^, eV
**1**	227, 280, 325	450	27.1	1.04; 1.43; 1.76	−2.28	−5.41	−2.09	3.32
**2**	243, 289, 341, 425, 465	621	0.5 (3.6)	0.76 low 0.96 low 1.10 low 1.41/1.34	−1.39/−1.31 −1.63/−1.53 −1.99/−1.92 −2.42	−5.13	−3.02	2.72

^a^ Values without parentheses obtained in air-saturated solutions; values in parentheses are for degassed solutions. ^b^ Electrochemical potentials are reported vs. Ag/AgCl/KCl_sat_. ^c^ The HOMO and LUMO energy levels for the complexes are estimated on the basis of the ferrocene energy level as internal standard (4.8 eV below the vacuum level): HOMO/LUMO = −(E_ox or red_−0.43 V)−4.8 eV, where the value 0.43 V for ferrocene/ferrocenium E_1/2_ vs. Ag/AgCl/KCl_sat_.

**Table 2:** Calculated stability constants for bimetallic complexes.

Complex	lgK11 (Calculated from Absorption Spectra)
**2**·Ba^2+^	5.4 ± 0.1
**2**·Pb^2+^	6.7 ± 0.3
**2**·Cd^2+^	5.0 ± 0.2

**Table 3:** The calculated emission lifetimes of **2**, **2**∙Pb^2+^, **2**∙Ba^2+^, **2**∙Cd^2+^.

Compound	Emission Lifetimes in Air (Relative Amplitude)	Emission Lifetimes in Degassed Solution (Relative Amplitude)
**1**	6.410 ± 0.003 ns (100%)	-
**2**	140.98 ± 0.24 ns (98.03%) 14.66 ± 0.23 ns (1.97%)	570.80 ± 2.73 ns (99.80%) 15.53 ± 1.65 ns (0.20%)
**2**∙Pb^2+^	161.83 ± 0.33 ns (100%)	566.78 ± 1.45 ns (100%)
**2**∙Ba^2+^	149.67 ± 0.20 ns (100%)	579.82 ± 1.47 ns (100%)
**2**∙Cd^2+^	151.32 ± 0.19 ns (100%)	477.68 ± 3.60 ns (100%)

“ns” means nanoseconds and “-“ indicates that no measurements were performed for this sample.

**Table 4:** The optimized structure and localization of 4 boundary MOs of **2**, **2**∙Pb^2+^.

MO	**2** 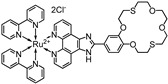	**2**∙Pb^2+^ 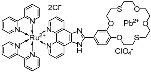
LUMO+1	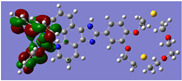	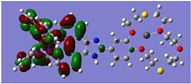
LUMO	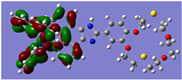	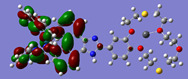
HOMO	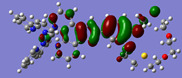	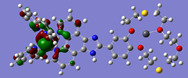
HOMO-1	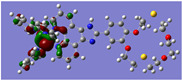	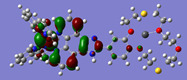

**Table 5:** Natural transition orbitals (NTOs) computed at the M06/def2-TZVP level of theory.

Natural Transition Orbitals			
Excitation	Complex	Occupied	Virtual
Around 445 nm	**2**	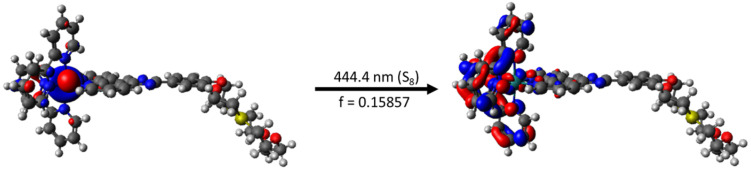	
	**2**∙Pb^2+^	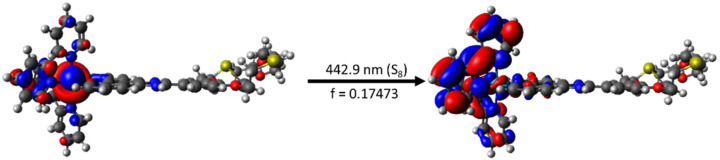	
Around 350 nm	**2**	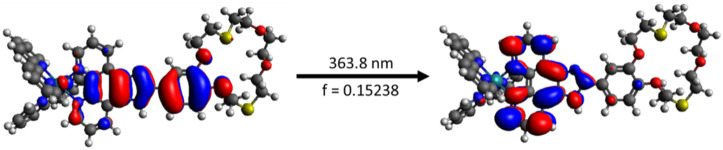	
	**2**∙Pb^2+^	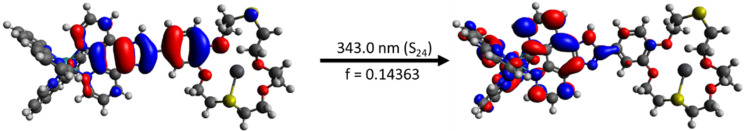	

## Data Availability

The original contributions presented in this study are included in the article/Supplementary Materials. Further inquiries can be directed to the corresponding author.

## Supplementary Materials

The following supporting information can be downloaded at: https://www.mdpi.com/article/10.3390/molecules31132213/s1, Figures S1 and S2: CVA of ligand **1** and complex **2**; Figures S3 and S4: Optical titration of complex **2** with cadmium and barium perchlorates; Table S1: Calculated stability constants for bimetallic complexes; Figures S5–S8: CVA titrations of complexes **2** and **3** with different metal cations; Figures S9–S11: NMR titration of complex **2** with different metal cations; Figures S12–S14, Table S2: EXAFS study of bimetallic complexes; Figures S15–S18: Emission spectra of complex **2** in air-saturated and degassed MeCN solutions with or without different metal cations; Figures S19–S27: emission decay of solution of **1** and **2** in air-saturated and degassed MeCN solutions with or without different metal cations; Figures S28 and S29: Comparison of electrochemical data with absorption spectroscopy results for **1** and **2**; Table S3: The optimized structure and localization of 4 boundary MOs of 2∙Ba^2+^ and 2∙Cd^2+^; Table S4: Details of the selected transitions in UV-visible spectra of complexes 2 and 2∙Pb^2+^ calculated using TDDFT; Figures S30 and S31: UV–vis absorption spectra of **2** and **2**∙Pb^2+^ in acetonitrile, along with the oscillator strengths (blue bars) calculated by TD-DFT (see [31,32,33,34]).

## Abbreviations

The following abbreviations are used in this manuscript:

MLCT	Metal-to-ligand charge transfer
ILCT	Intraligand charge transfer
ILLCT	Interligand-to-ligand charge transfer
MC	Metal-centered
DFT	Density functional theory
PET	Photoelectron transfer
CVA	Cyclic voltammetry
EXAFS	X-ray absorption fine structure
HOMO	Highest occupied molecular orbital
LUMO	Lowest unoccupied molecular orbital
ImPh	Imidazo-[4,5-f][1,10]-phenanthroline
bipy	2,2’-bipyridine
