# Supplementary material for: Switching Between ILCT and 3MLCT Excited States by Complex Formation in Ruthenium–Polypyridine Complex Containing Thiacrown-Ether Unit
Source: Molecules. 2026 Jun 24;31(13):2213. doi: 10.3390/molecules31132213 (PMC13363696; doi:10.3390/molecules31132213)
Supplement: Supplementary file 1 [file molecules-31-02213-s001.zip › molecules-4319733-supplementary.pdf]

Switching Between ILCT and ³MLCT Excited States by Complex Formation in Ruthenium–Polypyridine Complex Containing Thiacrown-Ether Unit

Sergey Tokarev [1,*], Anatoly Botezatu [1], Daria Kharkovskaya [1], Gediminas Jonusauskas [2], Yuri Fedorov [1] and Olga Fedorova [1]

[1] A.N. Nesmeyanov Institute of Organoelement Compounds, Russian Academy of Sciences, 119334 Moscow, Russia; botezatu@ineos.ac.ru (A.B.); daryakharkovskaya@yahoo.ca (D.K.); fedorov@ineos.ac.ru (Y.F.); fedorova@ineos.ac.ru (O.F.)
[2] Laboratoire Ondes et Matière d'Aquitaine–UMR CNRS 5798, University of Bordeaux, 351 cours de la Libération, 33405 Talence, France; gediminas.jonusauskas@u-bordeaux.fr
* Correspondence: tokarev@ineos.ac.ru

Table of contents

Physicochemical properties 1
Structure study of bimetallic complexes in solutions 3
Structure study of bimetallic complexes precipitates 9
Steady-state and time-resolved emission data 12
Comparison of electrochemical data with absorption spectroscopy results 19
DFT calculations 21

Physicochemical properties

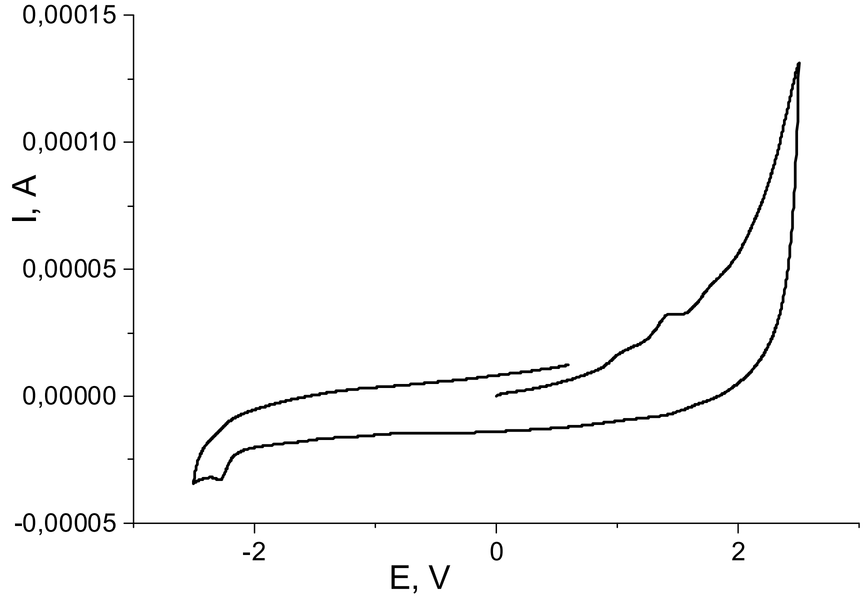

Figure S1. Cyclic voltammogram of $1,67\cdot10^{-4}$ M solution of ligand **1** in 0.1 M TBAHFP in MeCN at 200 mV/s. Oxidation potentials: 0,98; 1,34 V, reduction potential: -2,28 V vs Ag/AgCl reference electrode

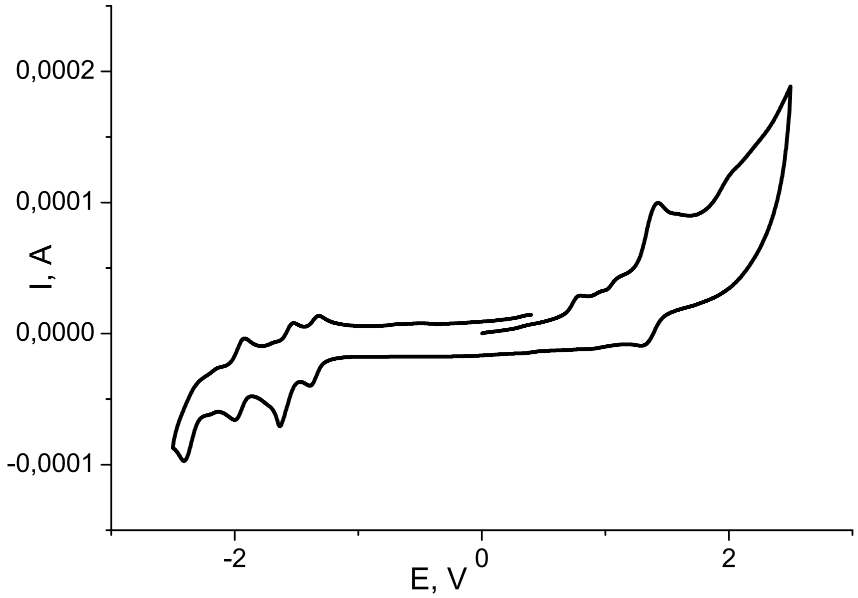

Figure S2. Cyclic voltammogram of 10^{-3} M solution of complex **2** in 0.1 M TBAHFP in MeCN at 200 mV/s.

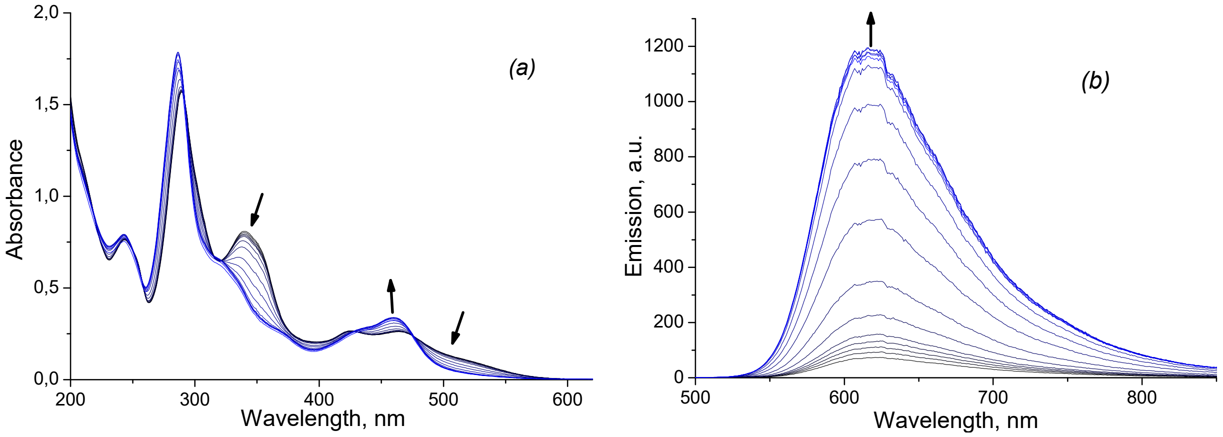

Figure S3: Electronic (*a*) absorption and (*b*) emission spectra of a MeCN solution of complex **2** at various concentrations of cadmium(II) perchlorate. The initial concentration of **2** $C_2=2\cdot10^{-5}$ $mol\cdot L^{-1}$, the concentration of salt varies in the range of $0 - 10^{-4}$ $mol\cdot L^{-1}$

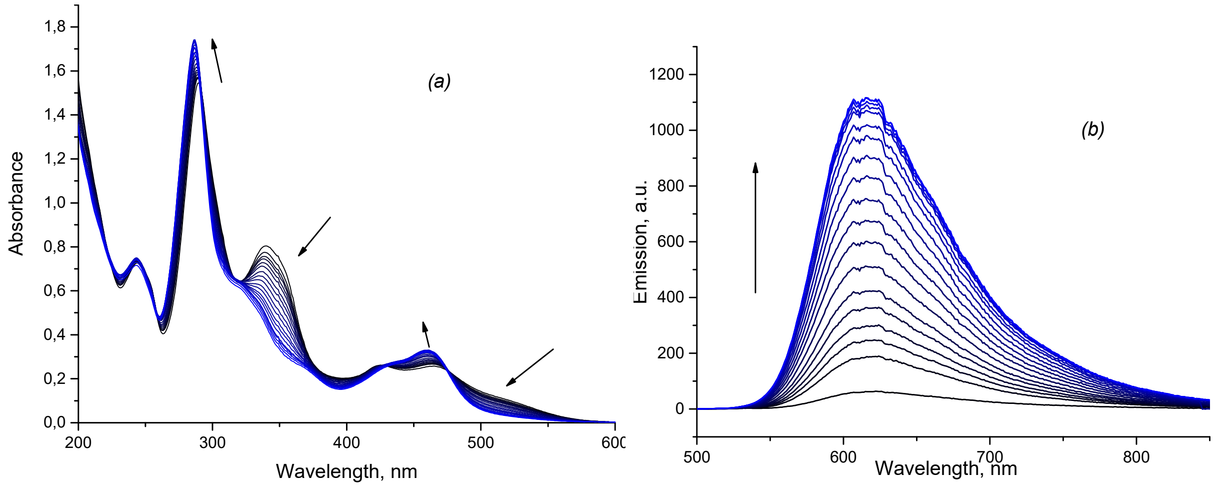

Figure S4: Electronic (a) absorption and (b) emission spectra of a MeCN solution of complex **2** at various concentrations of barium(II) perchlorate. The initial concentration of **2** $C_2=2\cdot10^{-5}$ $mol\cdot L^{-1}$, the concentration of salt varies in the range of $0 - 10^{-4}$ $mol\cdot L^{-1}$

Table S1. Calculated stability constants for bimetallic complexes.

M^{n+}	lgK_{11}
	Abs
Pb^{2+}	6.7±0.3
Cd^{2+}	5.0±0.2
Ba^{2+}	5.4±0.1

Structure study of bimetallic complexes in solutions

Upon addition of lead perchlorate to complex **2**, oxidation waves of sulfur atoms in the crown-ether fragment in the 0.7-1.2 V region vanish (Figure S5). This is due to the fact that the lone electron pairs of alkoxy-groups are electrostatically attracted to the lead cation and become less accessible for oxidation. Quasireversible ruthenium-centered oxidation slightly shifts to the 1.50/1.33 V. At the same time, in the -1.5 V region, a wave of reduction in the lead cation in a complex with crown-ether appears. It is noticeably shifted to the cathodic region relative to the reduction in Pb^{2+} in a free form (Figure S5b). Upon complexation, the electron density on the lead cation increases, which complicates its reduction. Addition of lead cation is accompanied by precipitation of a beige sediment. We believe that this is a precipitate of insoluble lead chloride. We were unable to find in the literature the solubility of this salt in acetonitrile; however, it is known to be extremely insoluble in water, ethanol, dioxane, and acetone. [1] To test this assumption, we synthesized the previously known ruthenium(II) complex **3** (Figure S6) [2], which is similar to **2**, but does not contain any substituents in the benzene ring of the ImPh ligand, so it is unable to coordinate metal cations. When adding portions of lead perchlorate to **3**, precipitation and the disappearance of the oxidation wave of Cl^- at 1.11 V are also observed. In the case of the addition of lead(II) perchlorate to complex **2,** there are two competitive processes for the lead cation: precipitation with chloride anion and complex formation with crown ether fragment.

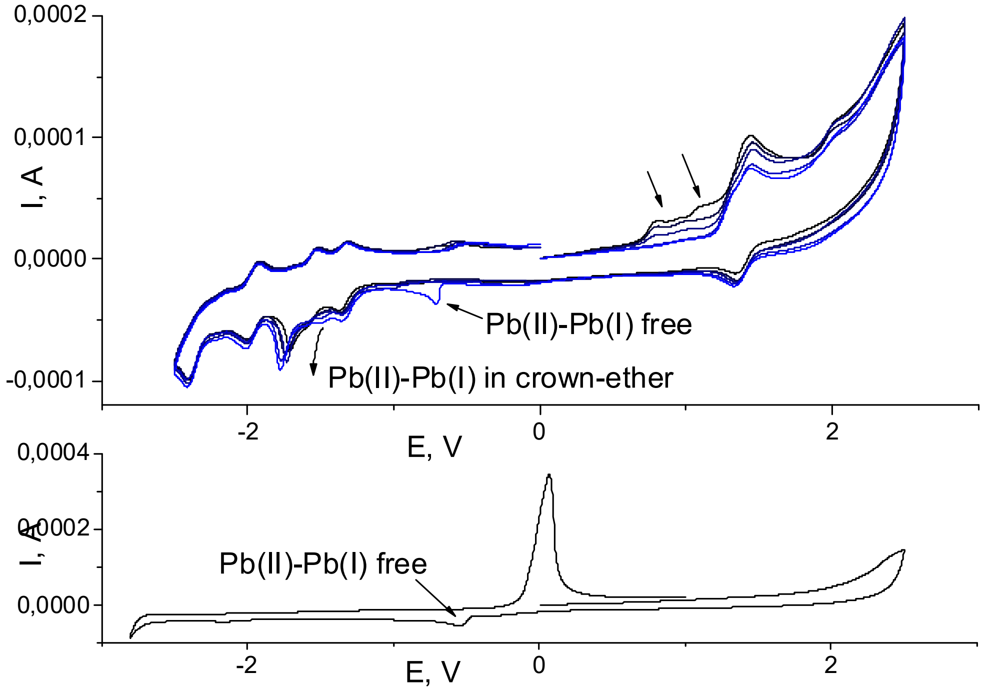

Figure S5 (a) Changes in cyclic voltammograms of complex **2** with the successive addition of lead perchlorate (molar ratio Ru^{2+}: Pb^{2+} 10:0; 10:5; 10:8; 10:10 and 10:15 from black to blue curve). The arrows show the potential shifts upon an increase in the lead cation concentration. **(b)** Cyclic voltammogram of lead perchlorate

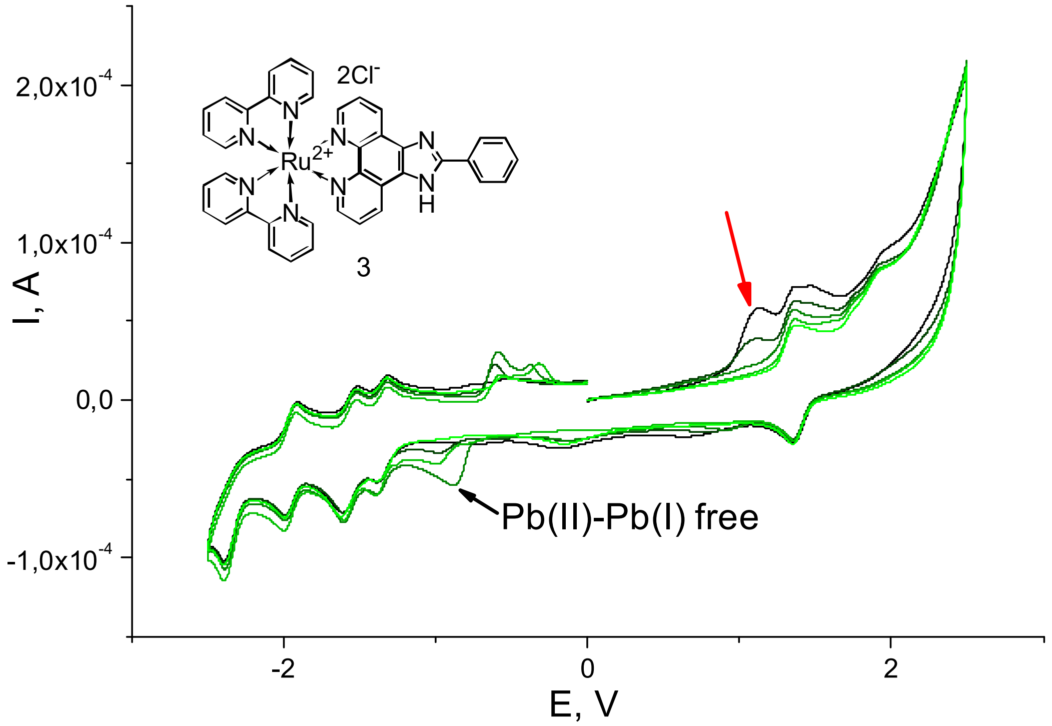

Figure S6. Changes in cyclic voltammograms of complex **3** with the successive addition of lead perchlorate (molar ratio Ru^{2+}:Pb^{2+} 10:0; 10:2; 10:5; 10:8; 10:10 from black to green curve). The red arrow shows the disappearance of chloride oxidation potential upon an increase in the lead cation concentration. Inset: complex **3** structure

In the case of the addition of cadmium(II) perchlorate to complex **2** (Figure S7), oxidation waves in the 0.7-1.2 V region also disappear, while sediment emerges. Although in this case, there is data on the very low solubility of cadmium(II) chloride in acetonitrile [3]. Thus, electrochemical studies suggest coordination of a second cation upon the dithia-18-crown-6 ether fragment of **2** in all cases studied, although this statement needs further proving. Another thing to be proved is that the ruthenium cation still remains bound to the 1,10-phenanthroline fragment.

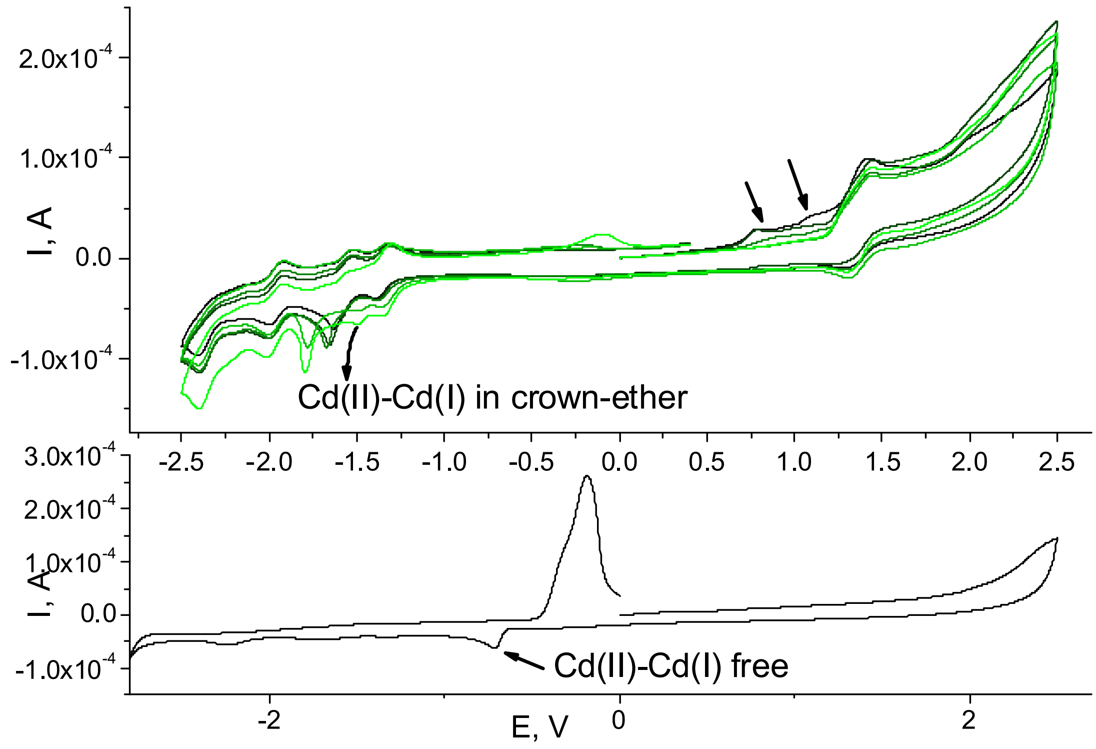

Figure S7. (a) Changes in cyclic voltammograms of complex **2** with the successive addition of cadmium (II) perchlorate (molar ratio Ru^{2+}: Cd^{2+} 10:0; 10:5; 10:8; 10:10; 10:15 from black to green curve). The arrows show the potential shifts upon an increase in the cadmium cation concentration. (b) Cyclic voltammogram of cadmium(II) perchlorate

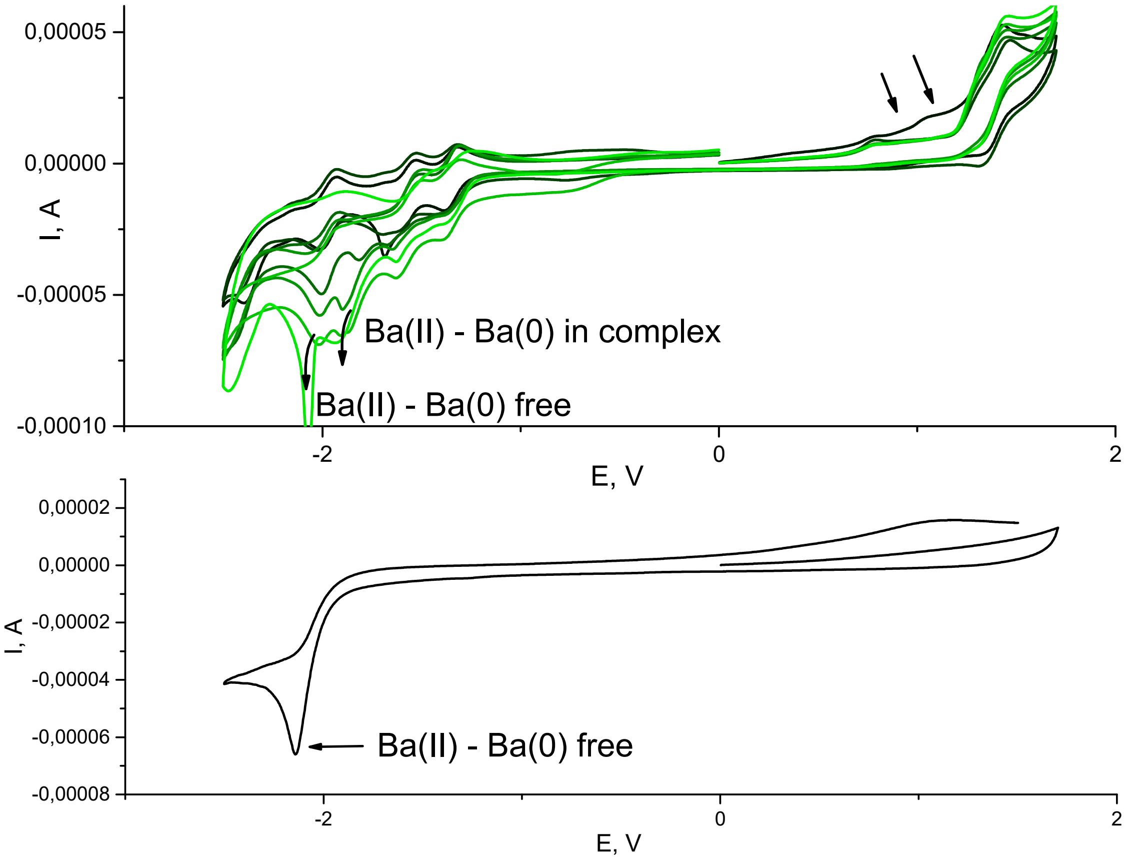

Figure S8. (a) Changes in cyclic voltammograms of complex **2** with the successive addition of barium (II) perchlorate (molar ratio Ru^{2+}: Cd^{2+} 10:0; 10:5; 10:8; 10:10; 10:15; 10:20 from black to green curve). The arrows show the potential shifts upon an increase in the barium cation concentration. (b) Cyclic voltammogram of barium(II) perchlorate

To confirm the coordination of second cations, the NMR spectra of ruthenium (II) and bimetallic complexes were studied. Bimetallic complexes were found to be poorly soluble in methanol, and the studies were carried out in d_3-MeCN. Signals of all crown-ether protons uniformly moved downfield upon the addition of one equivalent of lead(II) perchlorate (Figure S8). Aromatic protons of the ImPh ligand also experienced a downfield shift with a second cation addition, while bipy signals did not change. The lead (II) cation coordinates with all heteroatoms of the macrocyclic fragment.

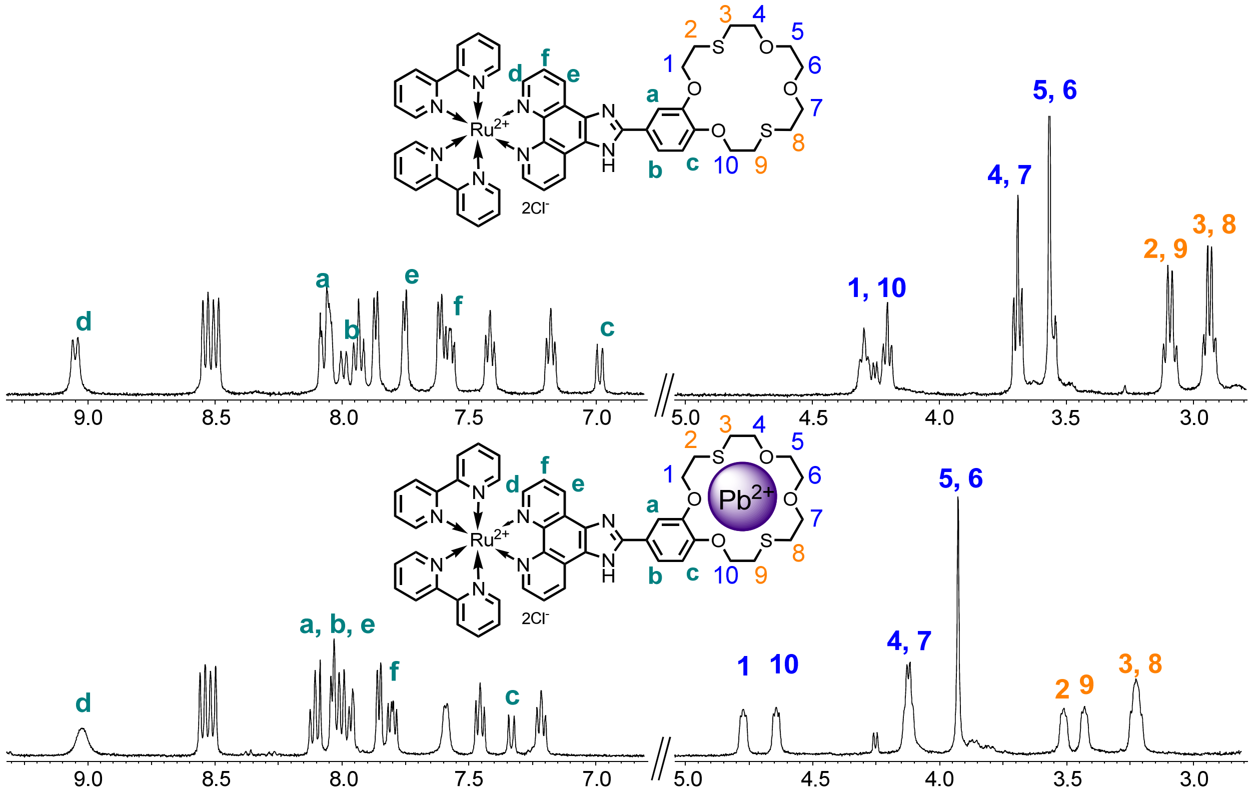

Figure S9. ^{1}H NMR spectral changes in complex **2** (upper spectrum) upon the addition of 1 equivalent (bottom spectrum) of lead(II) perchlorate in d-MeCN.

^{1}H NMR spectral changes upon the formation of a bimetallic complex with Cd^{2+} are similar to those with the lead(II) cation (Figure S8). Comparison of the NMR spectra of monoruthenium complex **2** and its derivative bimetallic complexes proves that the coordination of the second cation occurs upon the crown ether fragment of the molecule. From NMR data, we should also point out that only one coordination product of high stability forms in each case, since all NMR spectra of the bimetallic mixture contain only one set of signals that differs from the spectrum of **2**.

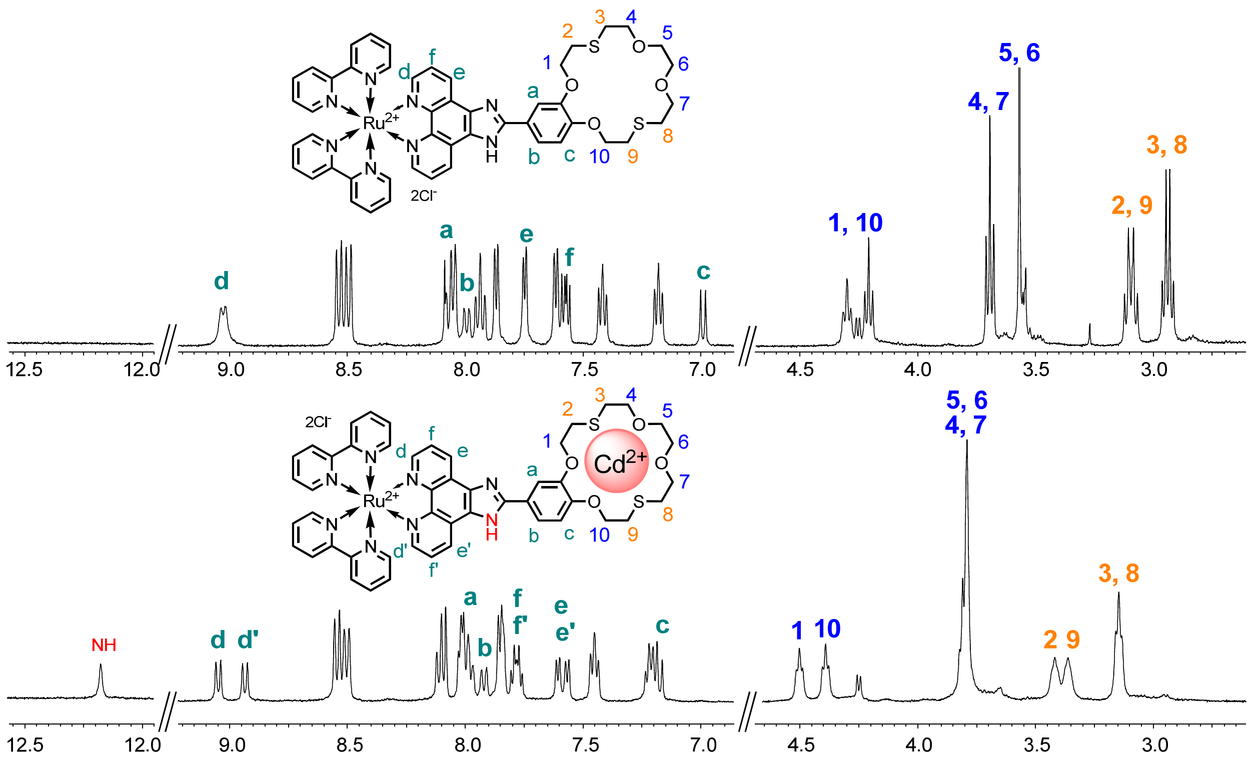

Figure S10. ^{1}H NMR spectral changes in complex **2** (upper spectrum) upon the addition of 1 equivalent (bottom spectrum) of cadmium(II) perchlorate in d-MeCN.

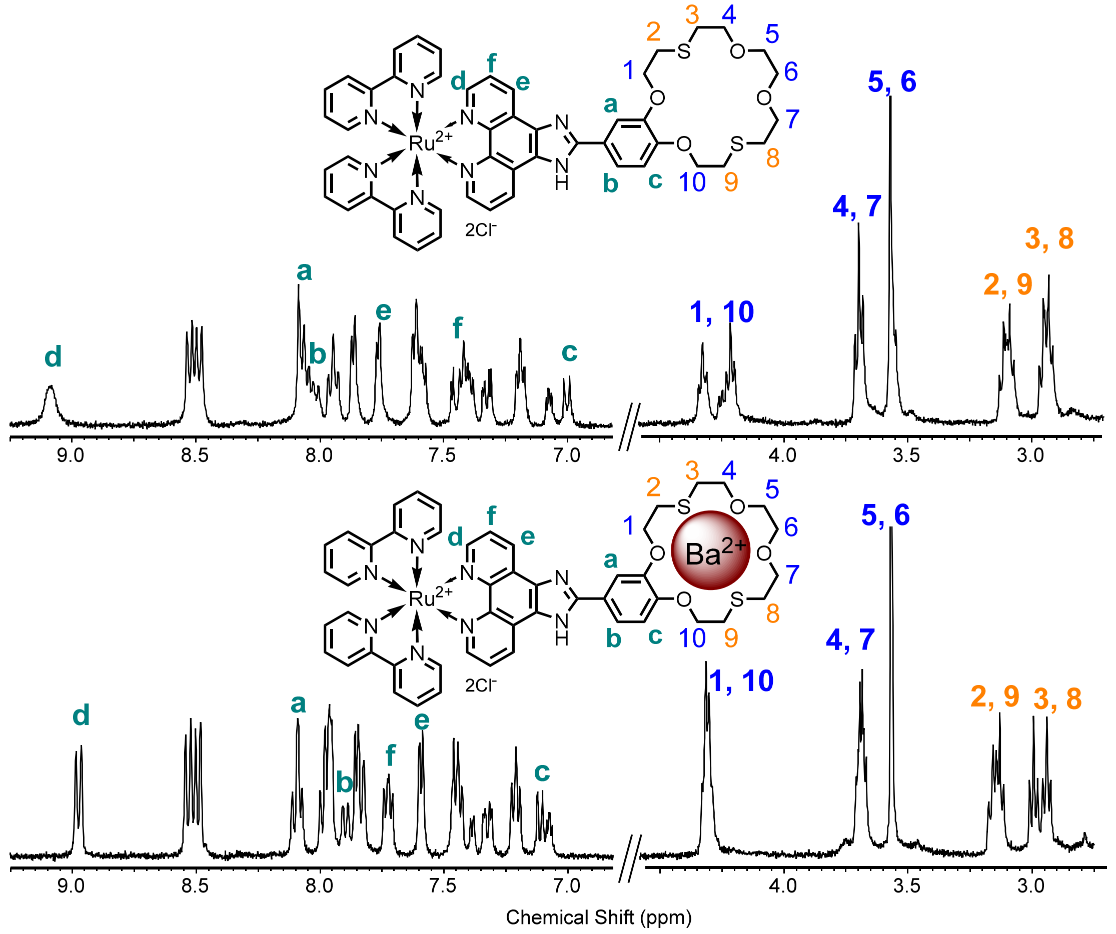

Figure S11. ^{1}H NMR spectral changes in complex **2** (upper spectrum) upon the addition of 1 equivalent (bottom spectrum) of barium(II) perchlorate in d-MeCN.

Structure study of bimetallic complex precipitates

Unfortunately, we were unable to obtain crystals of bimetallic complexes; however, bimetallic complexes were isolated in solid form. A concentrated solution of lead (II), barium (II) or cadmium (II) perchlorate was added to a solution of complex **2** in acetonitrile (1:1 molar ratio of Ru^{2+}:Me^{n+}), the mixture was stirred for 8 hours, and the bimetallic complexes were precipitated with diethyl ether. According to the observation by elemental analysis, bimetallic complexes were isolated in a mixture with the original ruthenium(II) complex in a ratio of 5:1 (bimetallic/monometallic). The technique was as follows.

To the 10^{-3} M solution of complex **2** in acetonitrile were added concentrated solutions of lead (II), barium (II) or cadmium (II) perchlorate. The resulting solution was stirred for 8 hours in the dark at room temperature. Acetonitrile was partially evaporated under reduced pressure, and a threefold volume of dry diethyl ether was added to precipitate a bright orange solid. The solid was isolated by filtration.

[Pb(**2**)]$(ClO_4)_2$. 19.6 mg of complex **2** were diluted in 20 ml of acetonitrile, and 211 mkl of $Pb(ClO_4)_2$ $8.9\cdot10^{-2}$ M solution was added. Yield: 19.2 mg (69%). Anal. Calcd for $C_{49}H_{46}Cl_4N_8O_{12}PbRuS2$: C, 40.50; H, 3.19; Pb, 14.26; Ru, 6.96; Found: C, 44.53; H, 3.86; Pb, 11.25; Ru, 6.76. Found fractions of elements correspond to a mixture of the initial ruthenium(II) complex and a bimetallic complex in a ratio of 1:5 with a small amount of solvent within the condensed phase. Anal. Calcd for 5·[Pb(**2**)$(CH_3CN)_4$]$(ClO_4)_2$+(**2**)$(CH_3CN)_4$: C, 44.24; H, 3.79; Pb, 11.09; Ru, 6.49.

[Ba(**2**)]ClO_4. 29.7 mg of complex **2** were diluted in 24 ml of acetonitrile, and 284 mkl of $Ba(ClO_4)_2$ acetonitrile 10^{-1} M solution was added. Yield: 22.8 mg (58%). Anal. Calcd. for $C_{49}H_{46}BaCl_4N_8O_{12}RuS_2$: C, 42.55; H, 3.35; Ba, 9.93; Ru, 7.31. Found: C, 45.49; H, 3.93; Ba, 8.20; Ru, 7.01. Found fractions of elements correspond to a mixture of the initial ruthenium(II) complex and a bimetallic complex in a ratio of 1:6 with a small amount of solvent inside the condensed phase. Anal. Calcd for 6·[Ba(**2**)$(CH_3CN)_3$]ClO_4+(**2**)$(CH_3CN)_3$: C, 45.30; H, 3.80; Ba, 8.07; Ru, 6.93.

[Cd(**2**)]ClO_4. 24.4 mg of complex **2** were diluted in 22 ml of acetonitrile, and 233 mkl of $Cd(ClO_4)_2$ acetonitrile 10^{-1} M solution was added. Yield: 23.4 mg (74%). Anal. Calcd. for $C_{49}H_{46}CdCl_4N8O_{12}RuS_2$: C, 43.33; H, 3.41; Cd, 8.28; Ru, 7.44. Found: C, 46.80; H, 4.04; Cd, 6.46; Ru, 6.92. Found fractions of elements correspond to a mixture of the initial ruthenium(II) complex and a bimetallic complex in a ratio of 1:5 with a small amount of solvent inside the condensed phase. Anal. Calcd for 5·[Cd(**2**)$(CH_3CN)_4$]ClO_4+(**2**)$(CH_3CN)_4$: C, 46.61; H, 3.99; Cd, 6.34; Ru, 6.84.

For the obtained bimetallic complexes **2**·Pb^{2+} and **2**·Cd^{2+} in solid form or acetonitrile solutions, the *K* X-ray absorption near-edge fine structure spectra of the Ru cation were analyzed (Figure S12-S14). In all cases, an octahedral environment of nitrogen atoms was found for the ruthenium (II) cation (Table S2). Thus, the second cation does not replace ruthenium at 1,10-phenanthroline.

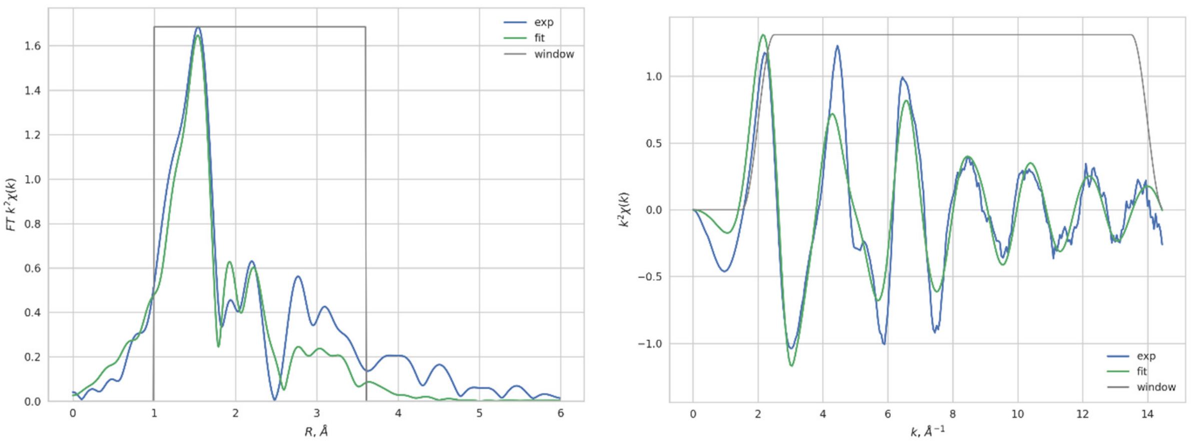

Figure S12. The fitted spectra for X-ray absorption of·k-space of Ru(II) for solid **2**·Pb^{2+}.

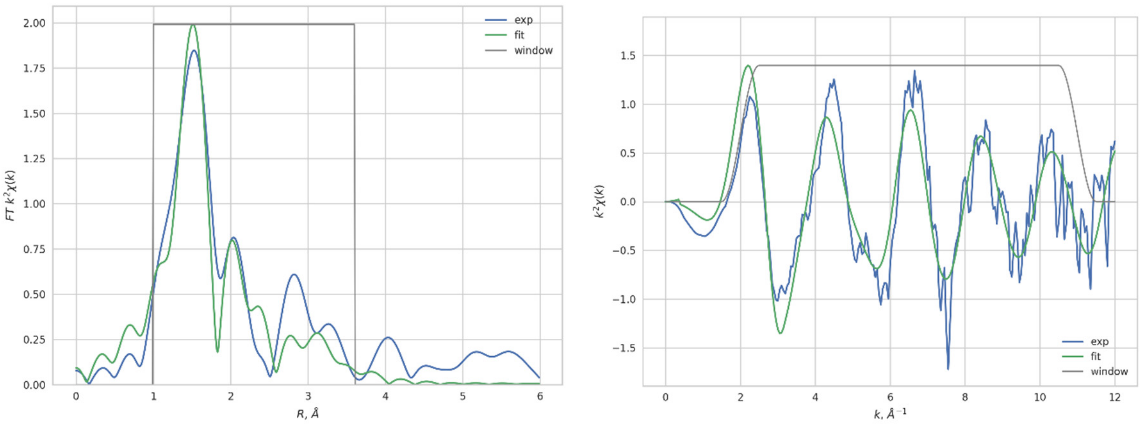

Figure S13. The fitted spectra for X-ray absorption of·k-space of Ru(II) for **2**·Pb^{2+} acetonitrile solution.

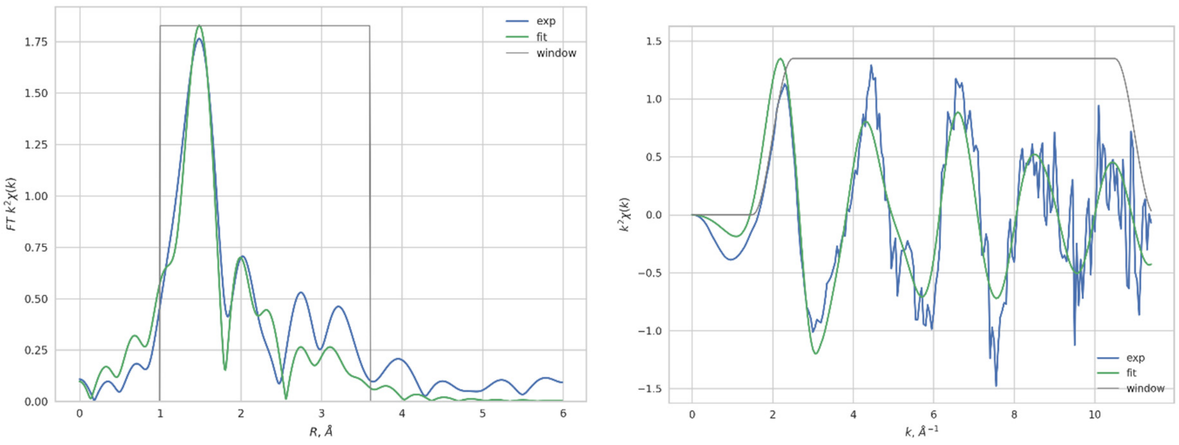

Figure S14. The fitted spectra for X-ray absorption of·k-space of Ru(II) for **2**·Cd^{2+} acetonitrile solution.

Table S2. Calculated parameters from EXAFS experiments.

Sample	R_f, %	Fit ranges	S_0^2	E_0	Path	N	R, Å	σ^2, 10^{-3} $Å^2$
	3.4	k: 2.0-11.0<br>R:1.0-3.6	0.70<br>±0.20	-0.6<br>±2.2	Ru-N	6.0	2.062<br>±0.016	0.4 ±2.9

**2**·Pb^{2+} acetonitrile solution					Ru-C	6.0	2.939 ±1.635	5.2 ±119.2
					Ru-C	6.0	3.003 ±1.641	5.2 ±119.2
					Ru-N-Ru-N-Ru	6.0	4.066 ±0.150	0.0 ±25.3
**2**·Cd^{2+} acetonitrile solution	2.9	k: 2.0-11.0 R:1.0-3.6	0.84 ±0.21	-0.4 ±2.3	Ru-N	6.0	2.053 ±0.018	0.8 ±2.5
					Ru-C	6.0	2.902 ±0.169	6.2 ±32.5
					Ru-C	6.0	3.027 ±0.172	6.2 ±32.5
					Ru-N-Ru-N-Ru	6.0	4.070 ±0.164	0.0 ±18.0
**2**·Pb^{2+} solid	2.2	k: 2.0-14.0 R:1.0-3.6	0.85 ±0.12	-0.5 ±1.3	Ru-N	6.0	2.060 ±0.009	2.1 ±1.3
					Ru-C	6.0	2.921 ±0.063	5.5 ±13.8
					Ru-C	6.0	3.043 ±0.078	5.5 ±13.8
					Ru-N-Ru-N-Ru	6.0	4.084 ±0.088	0.0 ±14.3

Steady-state and time-resolved emission data

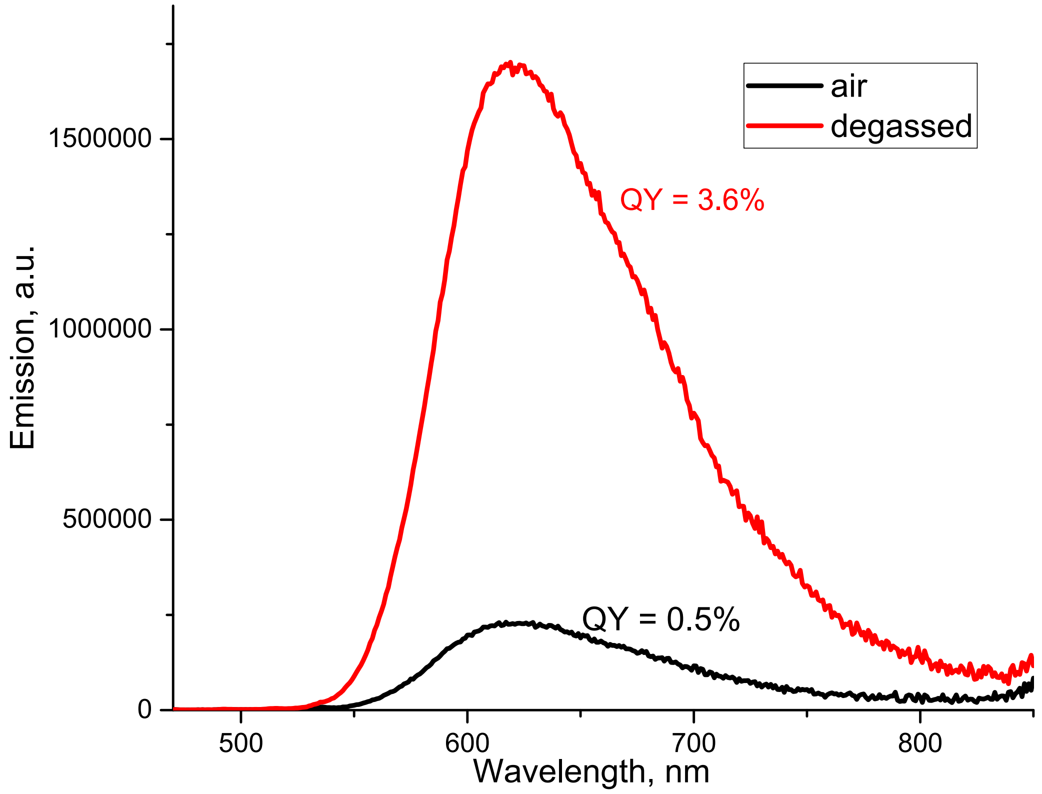

Figure S15: Emission spectra of complex **2** in air-saturated (black) and degassed (red) MeCN solution. Excitation at 460 nm

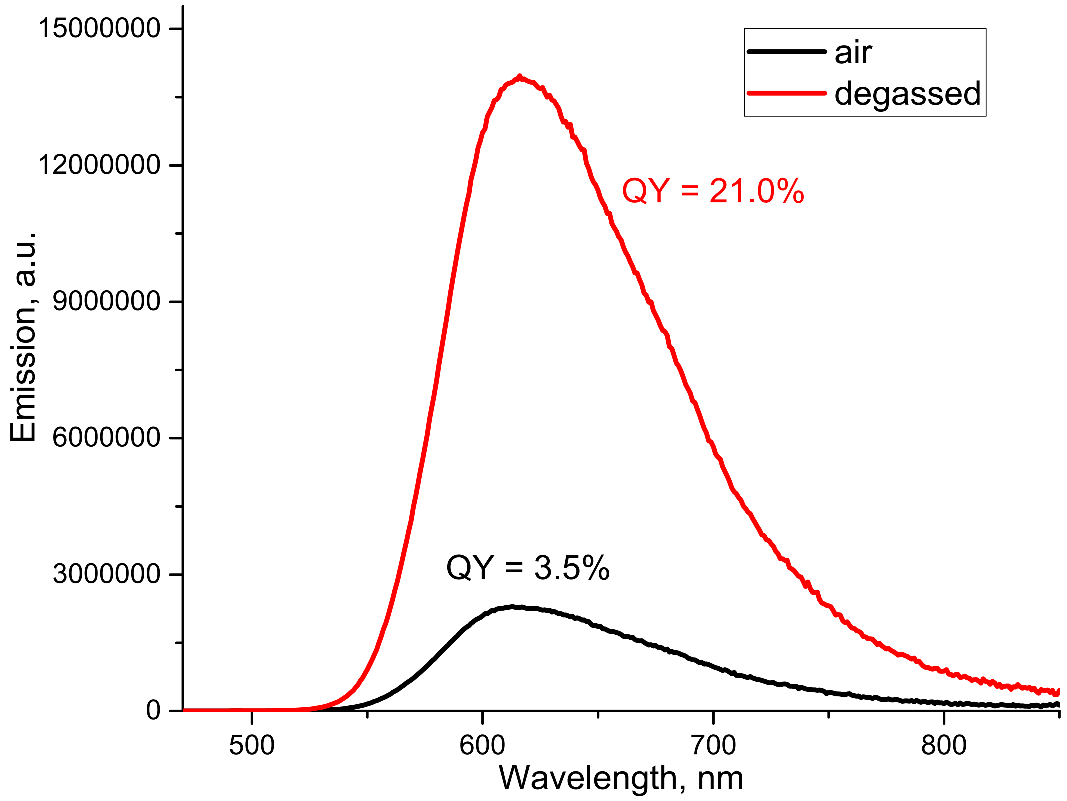

Figure S16: Emission spectra of a mixture of complex **2** with a 2-fold excess of $Pb(ClO_4)_2$ in air-saturated (black) and degassed (red) MeCN solution. Excitation at 460 nm

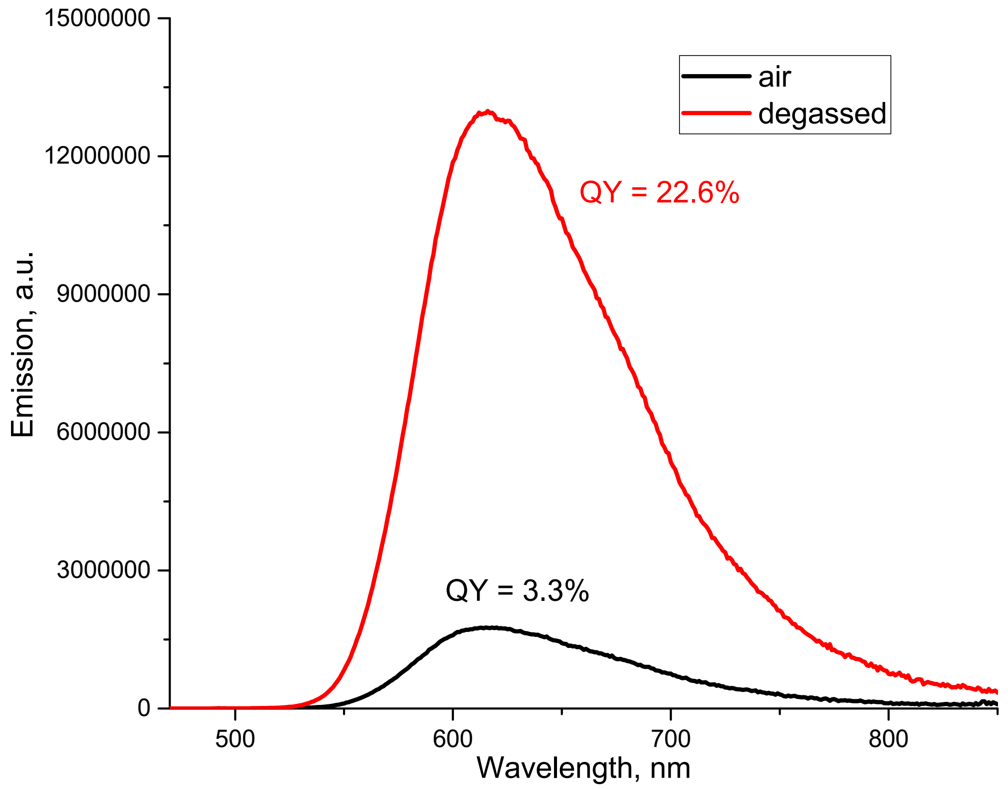

Figure S17: Emission spectra of a mixture of complex **2** with a 2-fold excess of $Ba(ClO_4)_2$ in air-saturated (black) and degassed (red) MeCN solution. Excitation at 460 nm

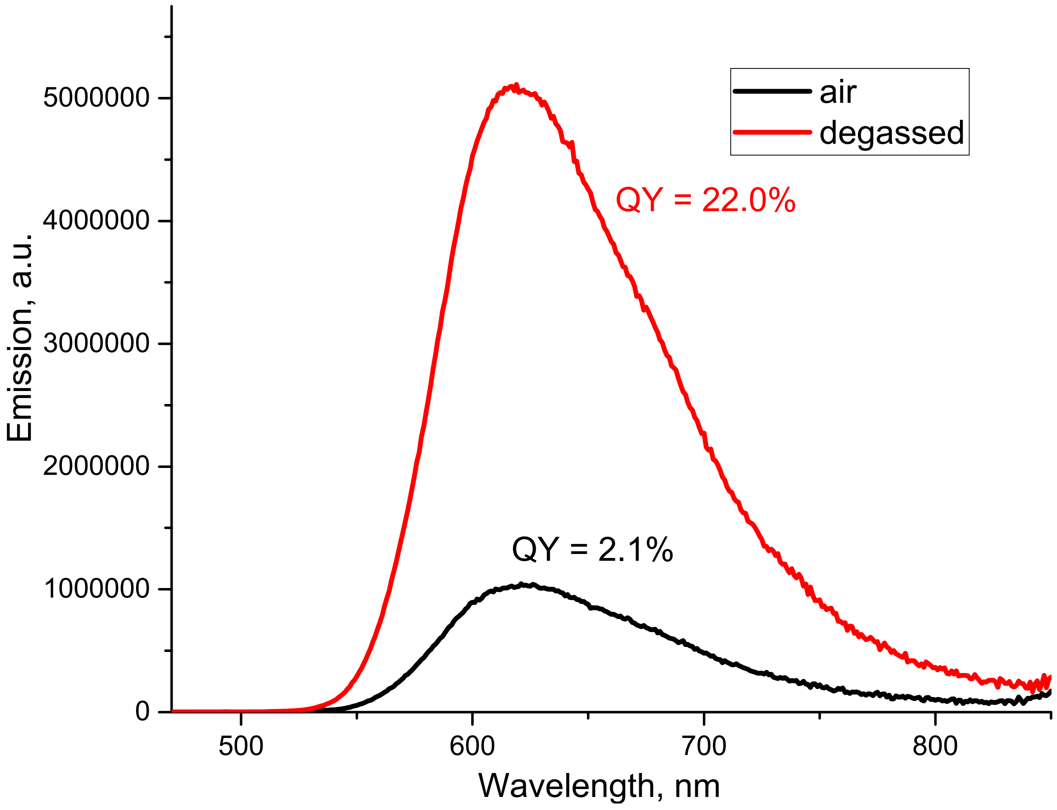

Figure S18: Emission spectra of a mixture of complex **2** with a 2-fold excess of $Cd(ClO_4)_2$ in air-saturated (black) and degassed (red) MeCN solution. Excitation at 460 nm

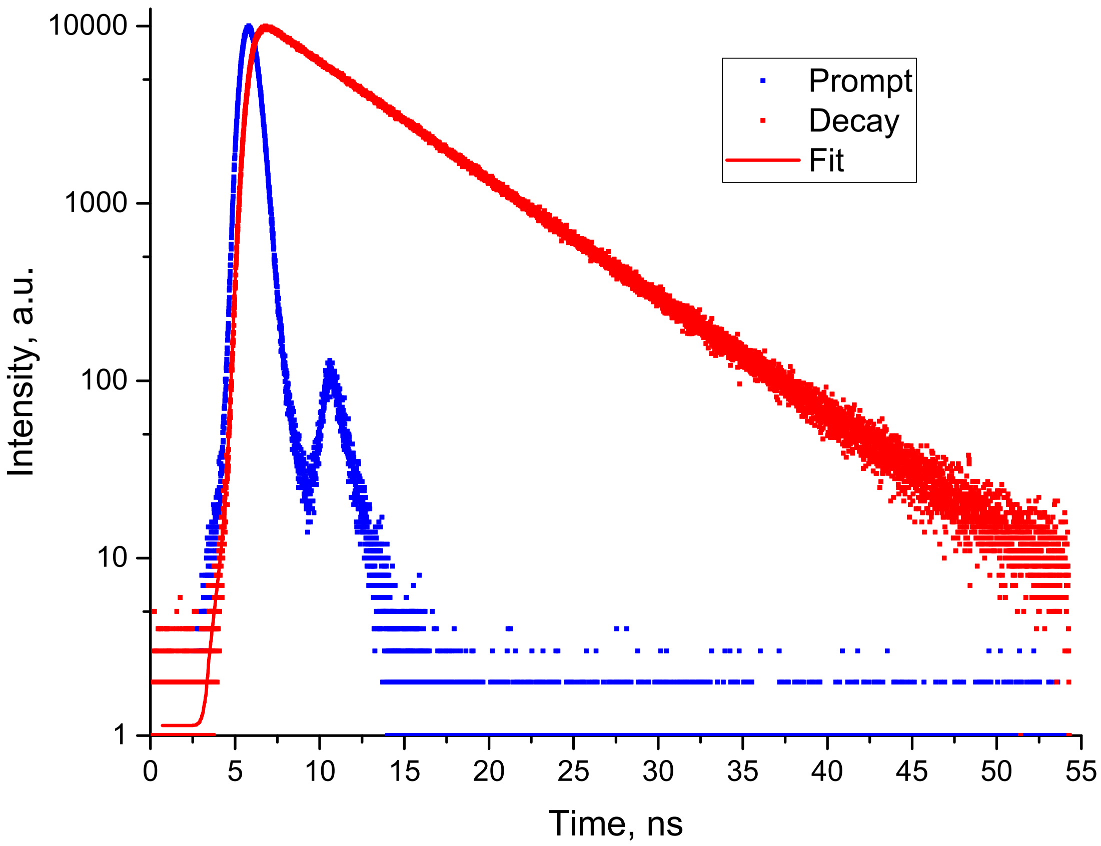

Figure S19. Emission decay of a solution of **1** in acetonitrile in air (red scatter) at 450 nm, 2 exponential fit (red line) and excitation flash of 310 nm (black scatter).

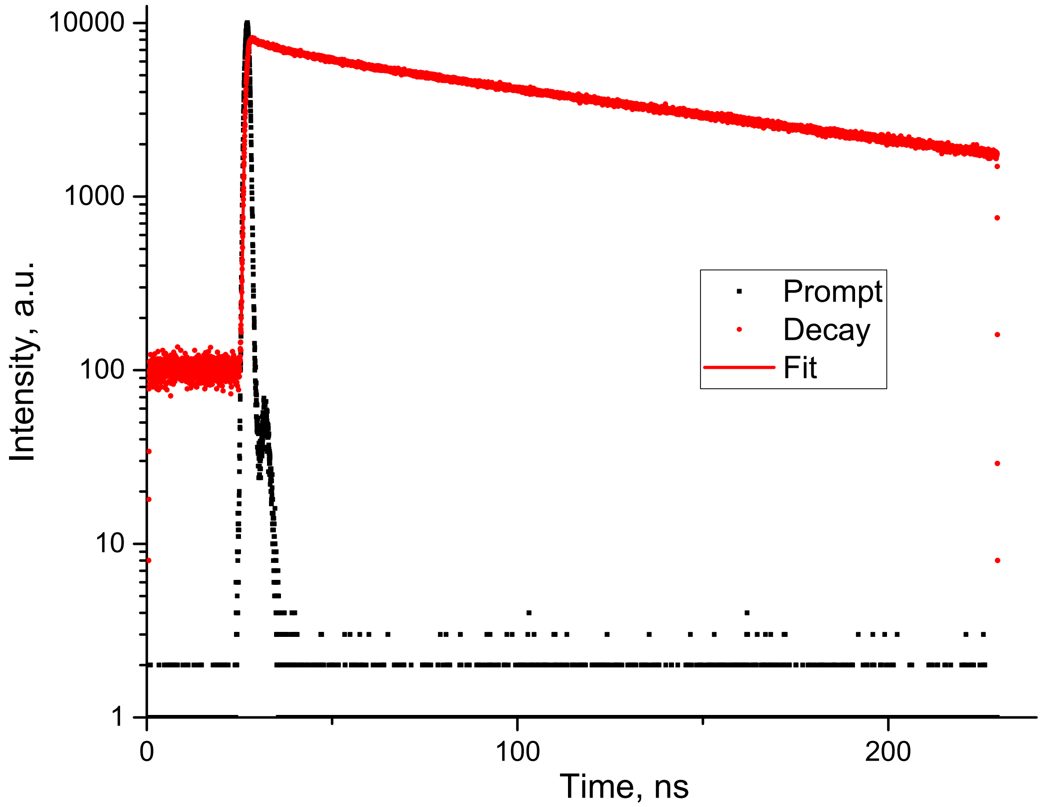

Figure S20. Emission decay of solution of **2** in acetonitrile in air (red scatter) at 620 nm, 2 exponential fit (red line) and excitation flash of 460 nm (black scatter).

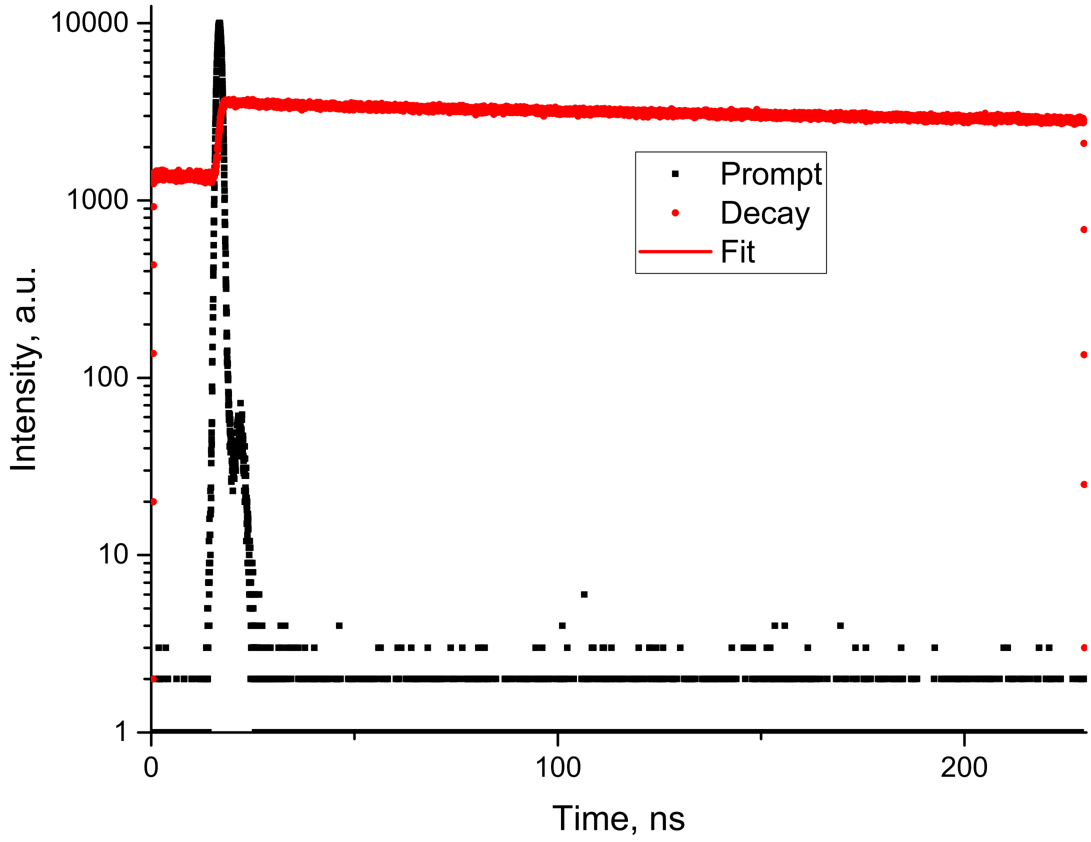

Figure S21. Emission decay of solution of **2** in the degassed acetonitrile (red scatter) at 620 nm, 2 exponential fit (red line) and excitation flash of 460 nm (black scatter).

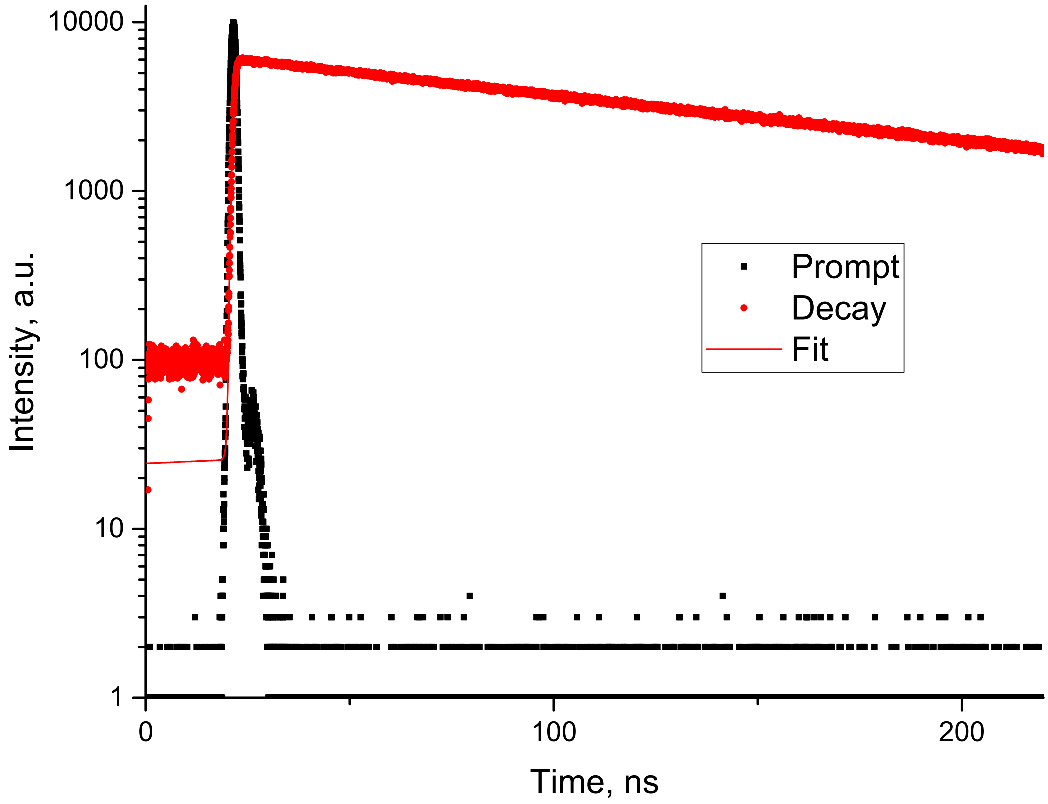

Figure S22. Emission decay of a solution of **2**·Pb^{2+} in acetonitrile in air (red scatter) at 620 nm, 2 exponential fit (red line) and excitation flash of 460 nm (black scatter).

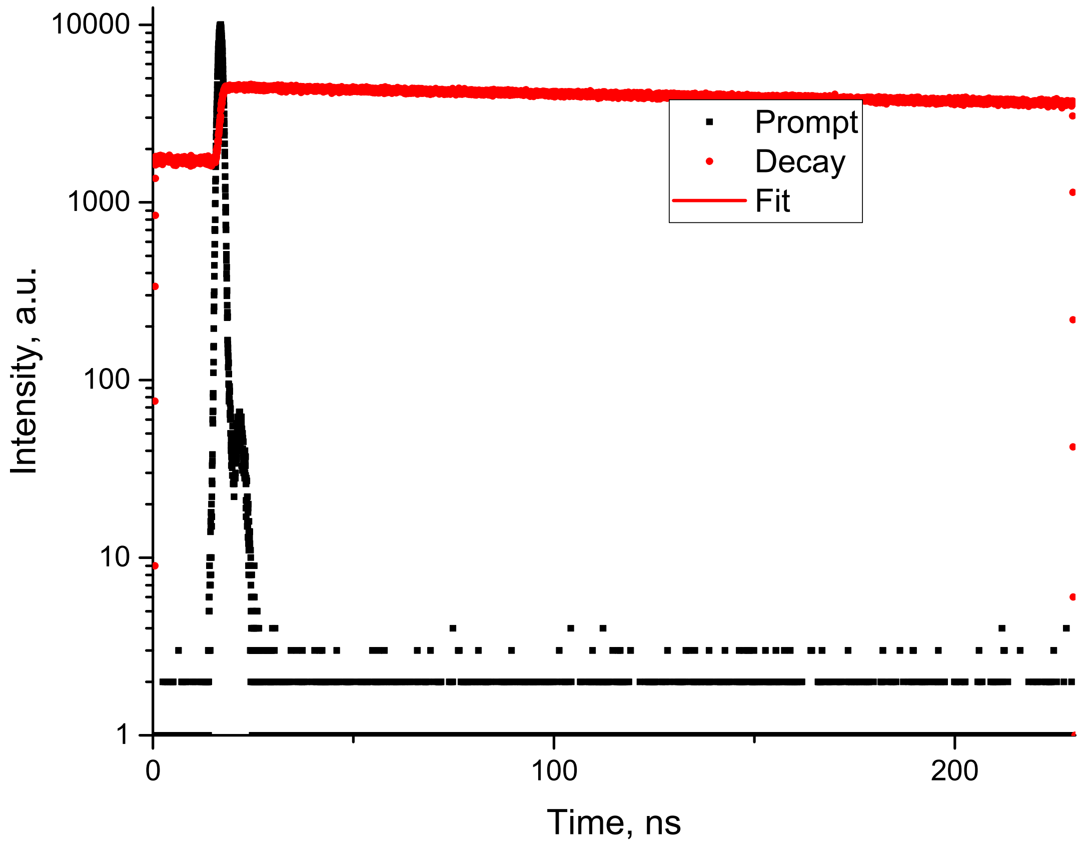

Figure S23. Emission decay of a solution of **2**·Pb^{2+} in the degassed acetonitrile (red scatter) at 620 nm, 2 exponential fit (red line) and excitation flash of 460 nm (black scatter).

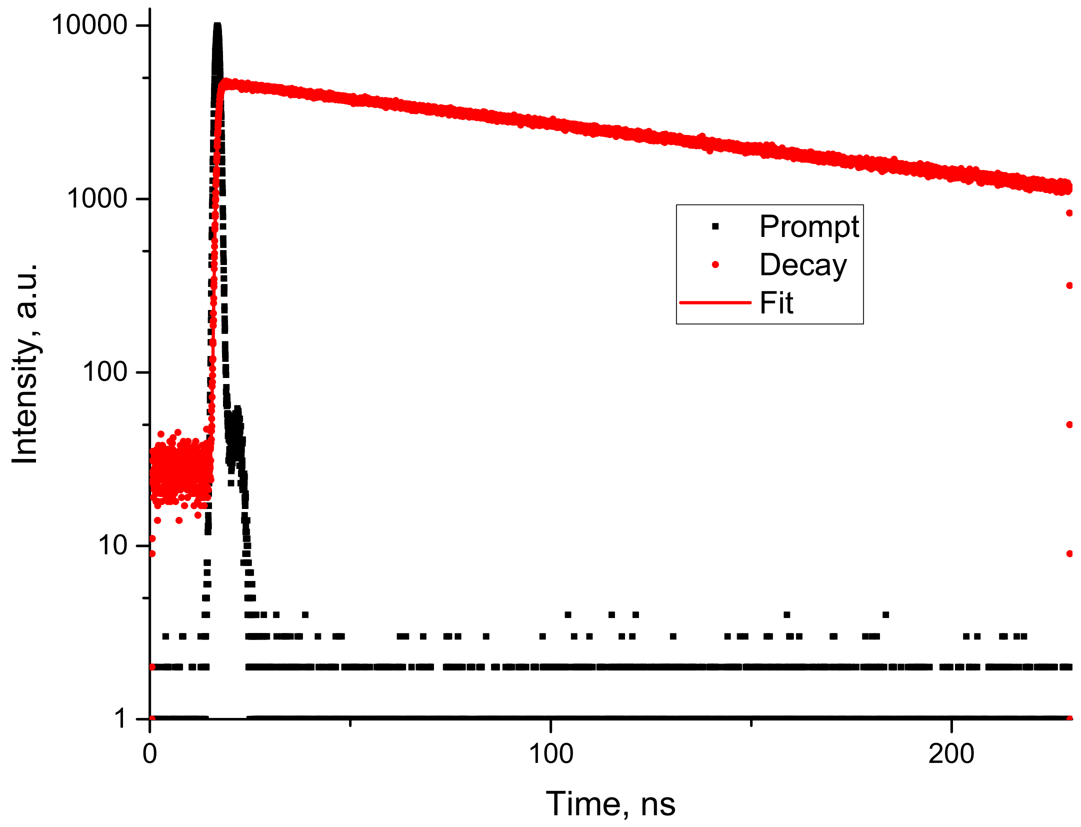

Figure S24. Emission decay of a solution of **2**·Cd^{2+} in acetonitrile in air (red scatter) at 620 nm, 2 exponential fit (red line) and excitation flash of 460 nm (black scatter).

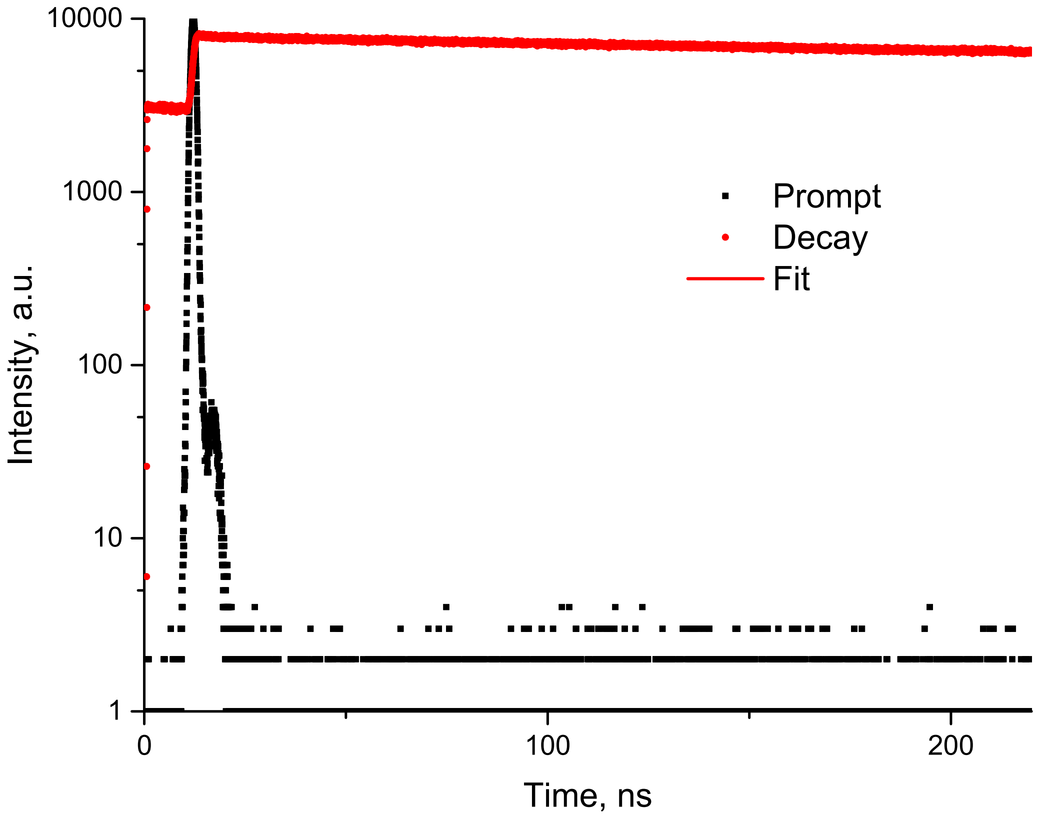

Figure S25. Emission decay of solution of **2**·Cd^{2+} in the degassed acetonitrile (red scatter) at 620 nm, 2 exponential fit (red line) and excitation flash of 460 nm (black scatter).

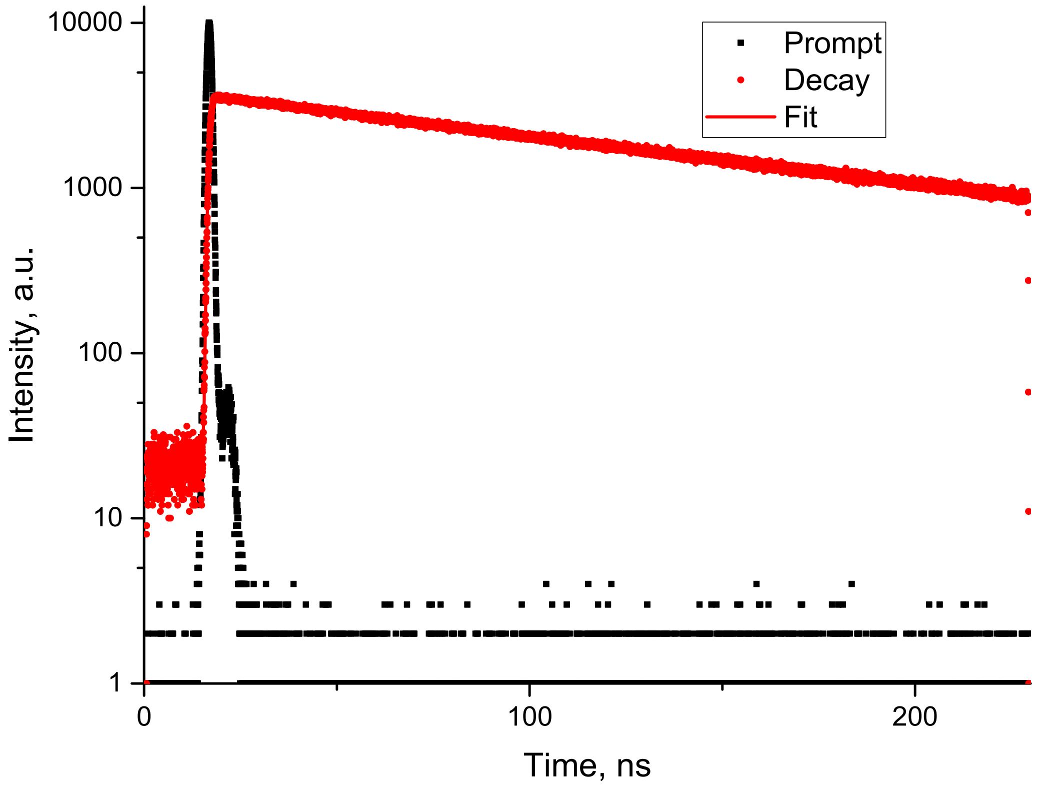

Figure S26. Emission decay of a solution of **2**·Ba^{2+} in acetonitrile in air (red scatter) at 620 nm, 2 exponential fit (red line) and excitation flash of 460 nm (black scatter).

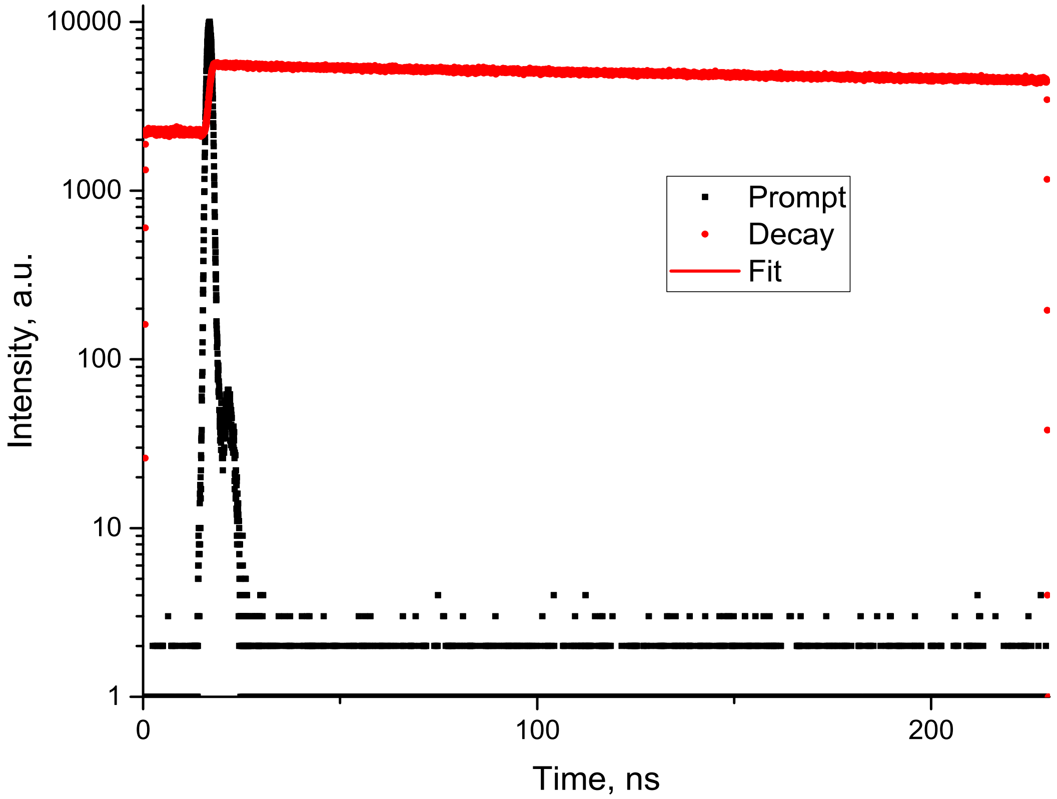

Figure S27. Emission decay of solution of **2**·Ba^{2+} in the degassed acetonitrile (red scatter) at 620 nm, 2 exponential fit (red line) and excitation flash of 460 nm (black scatter).

Comparison of electrochemical data with absorption spectroscopy results

To prove the hypothesis, electrochemical data were compared with steady-state absorption data. For ligand **1**, potentials are given vs. Ag/AgCl reference electrode: oxidation potentials are 1.04 V (HOMO), 1.43 V (HOMO-1) and 1.76 V (HOMO-2), reduction potential is -2.28 V (LUMO). The HOMO-LUMO gap is 3.32 eV (372 nm), the HOMO-1 – LUMO gap is 3.71 eV (333 nm), and the HOMO-2 – LUMO gap is 4.04 eV (306 nm). The transitions calculated from electrochemistry on the absorption spectrum are combined in Figure S28. As one can conclude, the correlation is quite accurate. There is one band at 320 nm, which is not attributed to an electrochemical signal. Perhaps, it corresponds to red-ox wave(s) of low intensity.

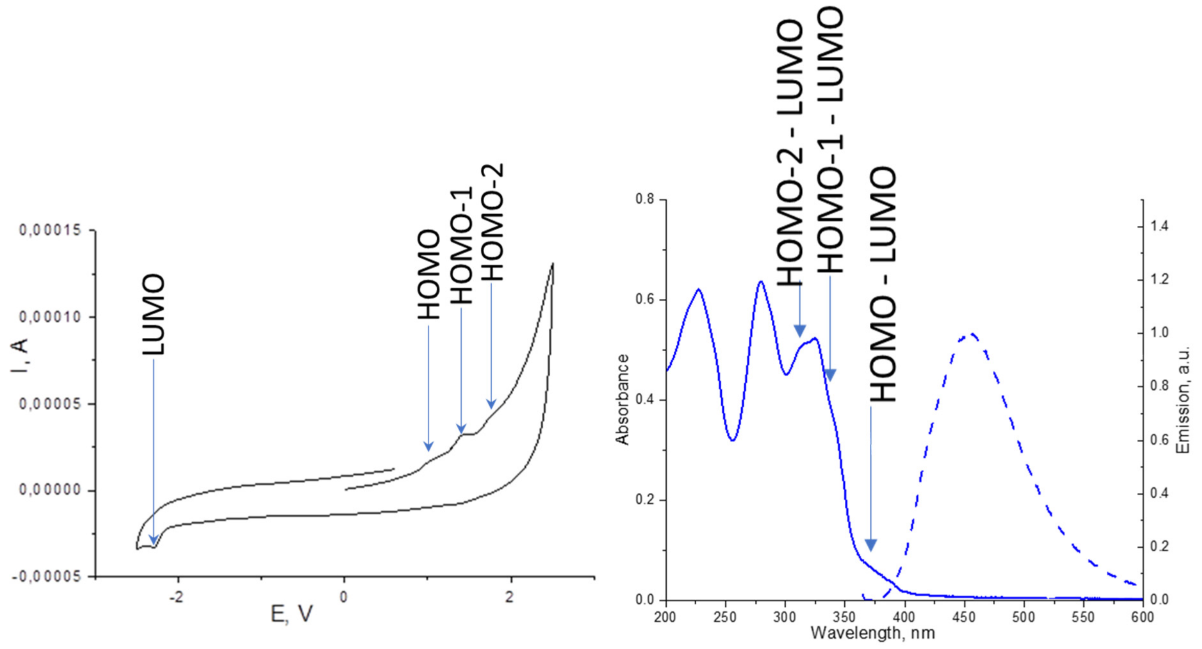

Figure S28. Voltammogram and steady-state absorption spectrum of ligand **1**.

The analog results for **2** are presented in Figure S29. The addition of two organic ligands, bipy, will produce additional redox waves; the Ru^{2+} will have its own wave; the oxidation waves of ligand **1** will be shifted since ruthenium(II) modifies a part of the electronic negativity of the **1,** making it easier to oxidize. This last effect corresponds to the increased charge transfer character of the **1** associated with Ru^{2+} and the shift in all steady-state absorption bands associated with **1** towards longer wavelengths. Oxidation potentials are 0.76 V (HOMO, **1**), 0.96 V (HOMO-1, **1**), 1.10 V (HOMO-2, **1**), 1.41 V ($Ru^{2+} \rightarrow Ru^{3+}$), possible at 1.9 V (HOMO-3, bipy); reduction potentials are 1.34 V ($Ru^{3+} \rightarrow Ru^{2+}$), -1.35 V (LUMO, bipy), -1.58 V (LUMO+1, bipy), -1.95 V (LUMO+2, bipy), -2.42 V (LUMO+3, **1**).

The lowest energy electronic transitions are located at 2.11 eV (HOMO → LUMO, 585 nm), 2.31 eV (HOMO-1 → LUMO, 534 nm), 2.34 eV (HOMO → LUMO+1, 527 nm), 2.45 eV (HOMO-2 → LUMO, 504 nm), 2.54 eV (HOMO-1 -> LUMO+1, 486 nm), 2.71 eV (HOMO → LUMO+2, 456 nm). All these transitions are Inter Ligand-Ligand Charge Transfer (ILLCT) transitions and usually possess a low oscillator strength since the overlap of molecular orbitals between ligands is weak.

The transitions associated with ligand **1** are: 3.18 eV (HOMO → LUMO+3, 388 nm), 3.38 eV (HOMO-1 → LUMO+3, 365 nm), 3.52 eV (HOMO-2 → LUMO+3, 351 nm). The oscillator strengths are high for Intra Ligand Charge transfer (ILCT) transitions, usually.

The transitions associated with bipy ligands are 3.25 eV (HOMO-3 → LUMO, 380 nm), 3.48 eV (HOMO-3 → LUMO+1, 355 nm), 3.85 eV (HOMO-3 → LUMO+2, 320 nm). These values may not be correct since the oxidation potential of BiPy was not correctly estimated. The overall tendency of the values of transition energies should be higher (the wavelengths should be shorter).

The transitions associated with Ru^{2+} are: 2.72 eV (Ru → LUMO, 453 nm), 2.95 eV (Ru → LUMO+1, 418 nm), 3.32 eV (Ru → LUMO+2, 371 nm), 3.79 eV (Ru → LUMO+3, 326 nm). These transitions are known as Metal to Ligand Charge Transfer (MLCT).

The calculations of electronic transitions from electrochemistry data are quite well reproduced in the steady-state absorption spectrum in Figure 6. The analysis of electrochemistry data compared with the steady-state absorption for complex **2** shows clearly that the lowest excited state is a singlet state corresponding to an interligand charge transfer transition. So, this is the first evidence of low-energy ILLCT transitions that overlap with MLCT transitions.

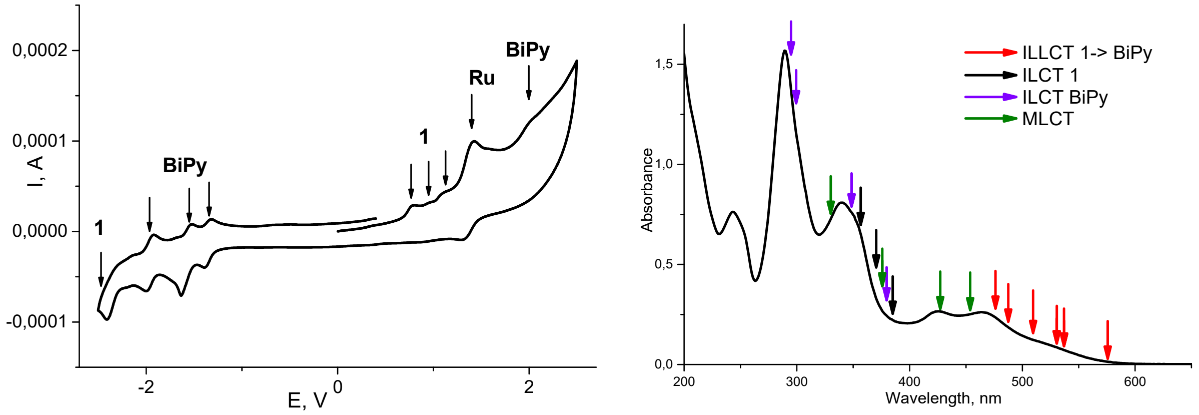

Figure S29. Voltammogram and steady-state absorption spectrum of complex **2**.

Finally, we compared the behavior of the monoruthenium complex **2** and bimetallic derivatives. The complexation with Pb^{2+}, Cd^{2+} and Ba^{2+} strongly affects the electrochemistry results (Figures S5, S7 and S8). The oxidation signal for the ligand **1** in complex disappears in the range 0.6-1.4 V. This result may be easily understood since the positive charge of the second cation fixes the electron excess on alkoxy groups, making it much more difficult to oxidize the ligand **1**.

DFT calculations

Geometry optimizations at the S0 minimum were performed without constraints at the B3LYP/3-21G level for atoms C, H, N, O, and B3LYP/LANL2DZ for Ru, Ba, Cd, Pb using Gaussian 09 software (revision D.01)[4] with corrections for solvation in acetonitrile (the PCM model). To find conformations with minimal energy, the conformational analysis was performed for all compounds. The geometry of the conformer with minimal energy was used for the following calculations. Selected molecular orbitals and their energies are given in Tables S3 and S4.

All calculations by the TDDFT method were carried out using the ORCA 6.0.1 software package with the def2-TZVPbasis set, using MO6 functionals. To model the effects of the solvent environment, the conductor-like polarizable continuum model was utilized, with acetonitrile chosen as the solvent to mimic the experimental conditions. A total of 80 singlet excited states were computed. For the analysis of excited-state character, Natural Transition Orbitals were generated, employing a threshold of 1×10^{-4} for the NTO eigenvalue summation.

Table S3. The optimized structure and localization of 4 boundary MOs of **2**·Ba^{2+} and **2**·Cd^{2+}

MO	**2**·Ba^{2+}	**2**·Cd^{2+}
LUMO+1	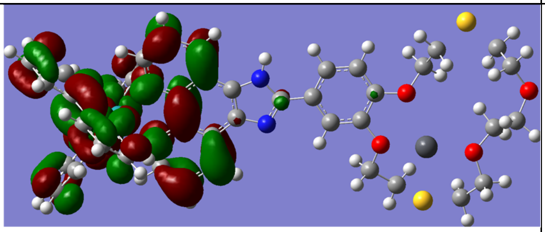	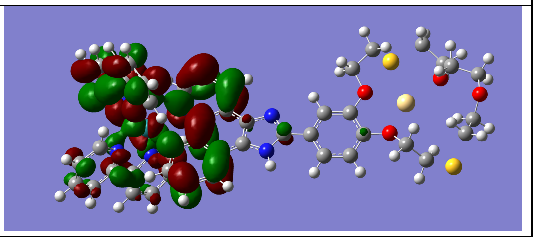
LUMO	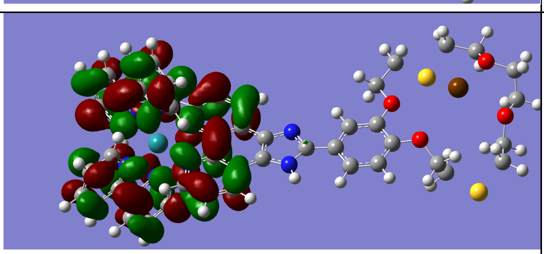	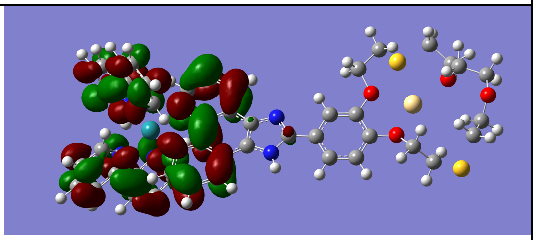
HOMO	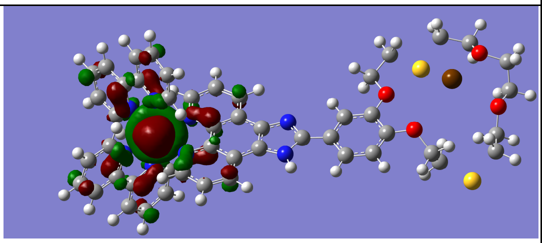	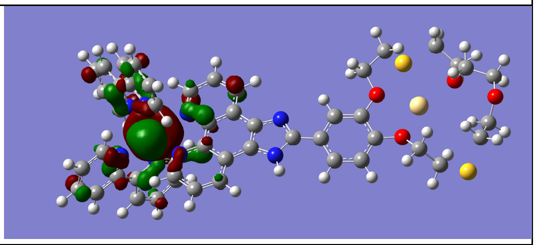
HOMO-1	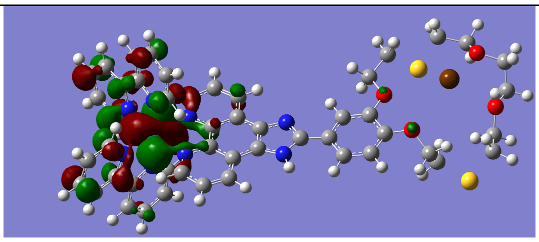	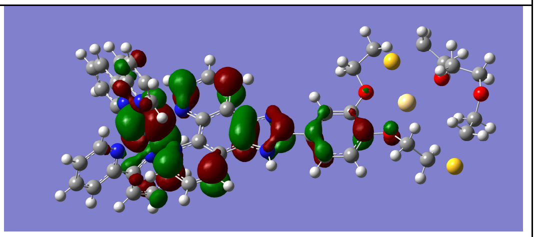

Cartesian coordinates and energies optimized at the B3LYP/3-21G level for atoms C, H, N, O, and B3LYP/LANL2DZ for Ru, Ba, Cd, Pb.

Complex 2

7	-1.228944000	0.313472000	-0.542215000
6	2.728258000	-1.021918000	-0.393314000
6	1.662240000	-1.893982000	-0.746258000
6	1.983069000	-3.231723000	-1.061887000
6	3.304279000	-3.642556000	-1.020148000
6	2.489358000	0.350444000	-0.048316000
6	1.183570000	0.892875000	-0.063833000
6	0.116384000	0.021500000	-0.434853000
6	0.357271000	-1.321834000	-0.755869000
7	4.034702000	-1.446108000	-0.369197000
6	3.399084000	2.403091000	0.624346000
6	2.127222000	2.997182000	0.626689000
6	1.015610000	2.247112000	0.282744000
6	-1.822641000	-0.829813000	-0.923021000
7	3.583492000	1.108158000	0.295757000
6	-3.247100000	-0.976941000	-1.177291000
6	-3.847461000	-2.204666000	-1.470565000
6	-5.221583000	-2.269188000	-1.712723000
6	-5.819052000	2.452902000	-0.706997000
8	-6.260039000	1.209981000	-1.362437000
6	-4.042968000	0.185774000	-1.124716000
16	-8.522126000	2.398425000	0.035981000
6	-7.041536000	3.357371000	-0.705133000
8	-11.400625000	2.514988000	1.018542000
6	-10.461035000	3.263060000	1.853791000
6	-9.414517000	3.843985000	0.914035000
8	-11.925088000	-0.406181000	1.147739000
6	-11.976407000	0.476373000	2.312147000
6	-12.438928000	1.838260000	1.800690000
6	-5.401967000	0.126513000	-1.374939000
6	-6.010120000	-1.123507000	-1.672913000

8	-7.366942000	-1.068565000	-1.919120000
6	-8.196728000	-2.276990000	-1.741124000
6	-8.309769000	-2.630305000	-0.255938000
16	-9.826699000	-3.741093000	0.046887000
6	-11.149423000	-2.368044000	0.056034000
6	-11.146510000	-1.617508000	1.386744000
7	-0.882731000	-1.846640000	-1.059812000
44	5.397755000	0.059315000	0.169669000
6	4.457605000	-0.413063000	3.065786000
6	6.409314000	-1.562421000	2.418844000
6	6.496939000	-2.151955000	3.681786000
6	5.539122000	-1.858770000	4.649506000
6	4.503573000	-0.975711000	4.335732000
6	7.366023000	-1.807645000	1.330207000
6	8.487534000	-2.631027000	1.448796000
7	5.390072000	-0.693710000	2.117909000
6	7.924100000	-1.332791000	-0.908206000
6	9.051565000	-2.142262000	-0.837291000
6	9.338565000	-2.802228000	0.359502000
7	7.089192000	-1.163212000	0.150393000
6	5.077404000	0.354875000	-2.881548000
6	6.406249000	1.987044000	-1.824820000
6	6.609376000	2.631504000	-3.046844000
6	6.030209000	2.117593000	-4.204626000
6	5.252653000	0.960865000	-4.119854000
6	6.972283000	2.448706000	-0.549437000
6	7.811500000	3.557975000	-0.424996000
7	5.640224000	0.850828000	-1.748456000
6	7.100260000	2.064184000	1.770681000
6	7.936149000	3.160795000	1.943436000
6	8.298656000	3.919776000	0.828715000
7	6.621033000	1.706193000	0.550468000
6	4.311102000	-2.730498000	-0.673085000
1	1.204462000	-3.932082000	-1.334722000
1	3.576763000	-4.661960000	-1.255812000

1	4.279264000	2.969594000	0.889875000
1	2.036179000	4.039700000	0.898931000
1	0.021520000	2.673978000	0.273330000
1	-3.270418000	-3.120364000	-1.503826000
1	-5.670029000	-3.228557000	-1.932151000
1	-5.003262000	2.917658000	-1.273059000
1	-5.483039000	2.235708000	0.312154000
1	-3.557880000	1.121450000	-0.885382000
1	-6.858095000	4.239898000	-0.089740000
1	-7.306158000	3.653572000	-1.719962000
1	-9.980673000	2.617832000	2.600281000
1	-10.978156000	4.079022000	2.380625000
1	-9.889183000	4.468398000	0.156803000
1	-8.670134000	4.413270000	1.473465000
1	-10.987071000	0.564261000	2.782301000
1	-12.682707000	0.097037000	3.063839000
1	-13.276708000	1.684068000	1.115985000
1	-12.769932000	2.462476000	2.641373000
1	-9.163080000	-1.973029000	-2.143666000
1	-7.816535000	-3.120098000	-2.324298000
1	-8.431830000	-1.720405000	0.333455000
1	-7.446583000	-3.187360000	0.108196000
1	-12.101989000	-2.864950000	-0.128195000
1	-10.936379000	-1.673833000	-0.758375000
1	-11.583533000	-2.239114000	2.179529000
1	-10.120451000	-1.356712000	1.681772000
1	-1.073376000	-2.797891000	-1.355329000
1	3.675895000	0.275583000	2.783188000
1	7.303198000	-2.834512000	3.907880000
1	5.600061000	-2.312297000	5.629850000
1	3.740877000	-0.723529000	5.059440000
1	8.695901000	-3.132427000	2.382487000
1	7.667085000	-0.806167000	-1.814711000
1	9.687836000	-2.248622000	-1.704991000
1	10.209774000	-3.438031000	0.444560000

1	4.485354000	-0.541062000	-2.773626000
1	7.211225000	3.527196000	-3.094568000
1	6.182613000	2.612287000	-5.154637000
1	4.786331000	0.530916000	-4.995501000
1	8.084320000	4.132544000	-1.298088000
1	6.796981000	1.452942000	2.606894000
1	8.293142000	3.408977000	2.933449000
1	8.949939000	4.777413000	0.932498000
1	5.348069000	-3.029107000	-0.642557000

Complex 2·Pb^{2+}

7	0.066124000	0.245874000	-0.081203000
6	-3.878889000	-1.065889000	0.316382000
6	-2.781468000	-1.939184000	0.543829000
6	-3.063504000	-3.267261000	0.925395000
6	-4.381425000	-3.668712000	1.066419000
6	-3.681647000	0.301477000	-0.078171000
6	-2.384413000	0.839940000	-0.229347000
6	-1.282369000	-0.035816000	0.006991000
6	-1.481903000	-1.375909000	0.366944000
7	-5.179327000	-1.482418000	0.462072000
6	-4.662928000	2.345322000	-0.669947000
6	-3.400510000	2.936889000	-0.829525000
6	-2.256049000	2.188319000	-0.611329000
6	0.697019000	-0.900297000	0.209762000
7	-4.808294000	1.055075000	-0.302930000
6	2.147121000	-1.044704000	0.231154000
6	2.798200000	-2.281976000	0.295335000
6	4.192403000	-2.343537000	0.288355000
6	4.576992000	2.518462000	0.544542000
8	5.135316000	1.196365000	0.099486000
6	2.907840000	0.135959000	0.152261000
16	6.954674000	3.648943000	-0.442291000
6	5.734501000	3.374035000	1.013645000
8	9.030036000	1.340140000	0.026854000
6	9.594898000	2.723704000	-0.117273000

6	8.630547000	3.721866000	0.494357000
8	11.704059000	-1.242503000	0.631552000
6	11.014538000	-0.169019000	-0.076496000
6	9.896377000	0.403252000	0.809360000
6	4.285695000	0.064851000	0.176315000
6	4.940163000	-1.174670000	0.241557000
8	6.351894000	-1.132629000	0.216995000
6	7.084655000	-2.156179000	1.067638000
6	7.774564000	-3.174950000	0.172859000
16	8.928104000	-4.266932000	1.227447000
6	10.291164000	-2.999472000	1.663435000
6	11.109542000	-2.576272000	0.430861000
7	-0.214973000	-1.909958000	0.489172000
44	-6.596046000	0.005106000	0.018257000
6	-5.901737000	-0.562064000	-2.931624000
6	-7.775972000	-1.705619000	-2.079771000
6	-7.954982000	-2.351932000	-3.304513000
6	-7.083364000	-2.089523000	-4.358882000
6	-6.041502000	-1.179410000	-4.168870000
6	-8.640284000	-1.912150000	-0.908870000
6	-9.764367000	-2.740592000	-0.905592000
7	-6.748830000	-0.813382000	-1.899220000
6	-9.016843000	-1.354593000	1.348367000
6	-10.142559000	-2.167954000	1.399412000
6	-10.523242000	-2.871847000	0.255041000
7	-8.272161000	-1.222806000	0.219505000
6	-6.010560000	0.394475000	3.018344000
6	-7.444387000	1.984326000	2.036898000
6	-7.548470000	2.661278000	3.253723000
6	-6.865554000	2.185257000	4.370403000
6	-6.084621000	1.033930000	4.250063000
6	-8.123915000	2.404479000	0.803324000
6	-8.981333000	3.503632000	0.721511000
7	-6.675965000	0.852678000	1.925868000
6	-8.454096000	1.948137000	-1.483426000

6	-9.314057000	3.032050000	-1.612929000
6	-9.582822000	3.822612000	-0.493629000
7	-7.863741000	1.631867000	-0.301033000
6	-5.419649000	-2.757859000	0.829645000
1	-2.260349000	-3.968634000	1.108590000
1	-4.625403000	-4.680380000	1.359395000
1	-5.568075000	2.908096000	-0.842472000
1	-3.341682000	3.975143000	-1.125609000
1	-1.267883000	2.612505000	-0.729559000
1	2.240051000	-3.208342000	0.321809000
1	4.681956000	-3.307192000	0.308904000
1	3.903771000	2.327970000	1.383654000
1	4.032787000	2.961321000	-0.290254000
1	2.383314000	1.077459000	0.075727000
1	5.372995000	4.355737000	1.317911000
1	6.282788000	2.893272000	1.823568000
1	9.776914000	2.909331000	-1.176701000
1	10.543685000	2.754464000	0.424298000
1	8.429197000	3.500382000	1.542286000
1	9.003733000	4.738565000	0.378910000
1	10.586833000	-0.520241000	-1.022919000
1	11.765845000	0.595121000	-0.289525000
1	9.226410000	-0.379541000	1.147377000
1	10.290234000	0.933557000	1.678138000
1	7.788179000	-1.571167000	1.654495000
1	6.358815000	-2.617605000	1.736931000
1	8.356417000	-2.698692000	-0.616347000
1	7.063470000	-3.868496000	-0.276688000
1	10.905678000	-3.518281000	2.400491000
1	9.834964000	-2.138873000	2.149409000
1	11.938654000	-3.270928000	0.278914000
1	10.490873000	-2.576964000	-0.473396000
1	0.009972000	-2.859884000	0.766979000
1	-5.110149000	0.147626000	-2.744918000
1	-8.764655000	-3.055052000	-3.434180000

1	-7.215273000	-2.587169000	-5.310412000
1	-5.344548000	-0.948663000	-4.962611000
1	-10.046891000	-3.275871000	-1.800266000
1	-8.688687000	-0.795036000	2.210967000
1	-10.705608000	-2.243106000	2.319385000
1	-11.396242000	-3.510788000	0.264642000
1	-5.419402000	-0.498481000	2.882946000
1	-8.154716000	3.552136000	3.329188000
1	-6.941362000	2.704751000	5.316363000
1	-5.539287000	0.633449000	5.093434000
1	-9.179551000	4.103285000	1.597843000
1	-8.220255000	1.314516000	-2.325318000
1	-9.761496000	3.246294000	-2.573661000
1	-10.249520000	4.671729000	-0.564278000
1	-6.453693000	-3.048909000	0.936525000
82	7.106313000	0.806879000	-1.105659000

Complex 2·Cd^{2+}

7	0.507363000	0.078212000	0.175430000
6	-3.485214000	-1.116678000	0.405554000
6	-2.423601000	-2.031075000	0.646657000
6	-2.761014000	-3.360022000	0.977514000
6	-4.095140000	-3.722847000	1.056989000
6	-3.231799000	0.254307000	0.060559000
6	-1.914115000	0.756524000	-0.019789000
6	-0.849702000	-0.161739000	0.224104000
6	-1.101495000	-1.506186000	0.529333000
7	-4.802769000	-1.496276000	0.486947000
6	-4.124837000	2.344815000	-0.507977000
6	-2.839850000	2.902237000	-0.592291000
6	-1.728979000	2.111452000	-0.352158000
6	1.096634000	-1.099079000	0.432738000
7	-4.324344000	1.048549000	-0.191443000
6	2.543363000	-1.284896000	0.424149000
6	3.171983000	-2.534236000	0.379813000
6	4.563003000	-2.612886000	0.269115000

6	4.969167000	2.271118000	0.437040000
8	5.548946000	0.913898000	0.239299000
6	3.322940000	-0.114728000	0.385569000
16	6.900688000	3.179419000	-1.370694000
6	6.112276000	3.272105000	0.380258000
8	9.560506000	1.634640000	-0.520329000
6	9.706148000	2.819641000	-1.408132000
6	8.664292000	3.882038000	-1.071428000
8	11.746828000	-0.173723000	1.298014000
6	11.689877000	1.179519000	0.753354000
6	10.277243000	1.781810000	0.790708000
6	4.698713000	-0.198124000	0.302158000
6	5.329215000	-1.456116000	0.225346000
8	6.721930000	-1.440510000	0.026077000
6	7.613874000	-2.445355000	0.718375000
6	8.560299000	-3.014988000	-0.330629000
16	10.056481000	-3.853237000	0.495267000
6	10.869065000	-2.360092000	1.360612000
6	11.241844000	-1.225868000	0.407011000
7	0.145934000	-2.086381000	0.658429000
44	-6.154428000	0.045335000	0.010556000
6	-5.331576000	-0.471200000	-2.916606000
6	-7.280671000	-1.573876000	-2.187159000
6	-7.422463000	-2.178732000	-3.437762000
6	-6.493051000	-1.917486000	-4.441742000
6	-5.431866000	-1.049377000	-4.176458000
6	-8.206778000	-1.783274000	-1.064904000
6	-9.354532000	-2.575329000	-1.138563000
7	-6.234671000	-0.722316000	-1.932639000
6	-8.679398000	-1.268351000	1.184220000
6	-9.831279000	-2.045369000	1.158257000
6	-10.175009000	-2.709608000	-0.021023000
7	-7.874516000	-1.134249000	0.097815000
6	-5.726901000	0.344868000	3.045247000
6	-7.030710000	2.018454000	2.021762000

6	-7.169708000	2.677097000	3.245089000
6	-6.571234000	2.148823000	4.386504000
6	-5.839467000	0.963838000	4.284521000
6	-7.623440000	2.490786000	0.762561000
6	-8.419272000	3.633385000	0.655098000
7	-6.308711000	0.855105000	1.928541000
6	-7.854724000	2.085528000	-1.546120000
6	-8.650614000	3.213986000	-1.701574000
6	-8.937877000	4.001003000	-0.584320000
7	-7.343773000	1.722584000	-0.340243000
6	-5.095566000	-2.773614000	0.806526000
1	-1.986721000	-4.091335000	1.169276000
1	-4.381365000	-4.734154000	1.310382000
1	-5.004133000	2.940576000	-0.702236000
1	-2.737579000	3.947217000	-0.850607000
1	-0.724126000	2.507042000	-0.416570000
1	2.598402000	-3.451747000	0.387825000
1	5.037060000	-3.581670000	0.188951000
1	4.493842000	2.305790000	1.421198000
1	4.229776000	2.461818000	-0.343980000
1	2.809790000	0.835171000	0.410559000
1	5.726659000	4.281868000	0.516796000
1	6.877504000	3.047702000	1.122752000
1	9.585820000	2.446380000	-2.426090000
1	10.714024000	3.227903000	-1.289655000
1	8.714998000	4.203551000	-0.030324000
1	8.767438000	4.743567000	-1.730306000
1	12.092846000	1.236431000	-0.265770000
1	12.332911000	1.763666000	1.416784000
1	9.675700000	1.256739000	1.533781000
1	10.312979000	2.845645000	1.039463000
1	8.133297000	-1.884151000	1.494675000
1	6.993433000	-3.217868000	1.170408000
1	8.937738000	-2.244969000	-1.002406000
1	8.083321000	-3.798804000	-0.919617000

1	11.760997000	-2.778405000	1.829468000
1	10.205361000	-1.983287000	2.138684000
1	12.009064000	-1.542667000	-0.308953000
1	10.367476000	-0.874932000	-0.151131000
1	0.333436000	-3.053640000	0.901650000
1	-4.528166000	0.207399000	-2.672931000
1	-8.248390000	-2.848902000	-3.626332000
1	-6.595885000	-2.383645000	-5.412609000
1	-4.690682000	-0.820664000	-4.929708000
1	-9.607655000	-3.079856000	-2.059474000
1	-8.378485000	-0.740119000	2.076002000
1	-10.442644000	-2.123210000	2.046623000
1	-11.066789000	-3.320089000	-0.071024000
1	-5.172017000	-0.572727000	2.921294000
1	-7.737026000	3.594296000	3.306672000
1	-6.674205000	2.654139000	5.337569000
1	-5.360816000	0.522353000	5.147668000
1	-8.633706000	4.229359000	1.530095000
1	-7.609090000	1.452647000	-2.385176000
1	-9.034732000	3.465181000	-2.680631000
1	-9.555559000	4.884553000	-0.675278000
1	-6.141161000	-3.036527000	0.862834000
48	7.615242000	0.524676000	-0.804608000

Complex 2·Ba^{2+}

7	-0.261269000	-0.096822000	0.105260000
6	3.761303000	-1.059677000	-0.475135000
6	2.720907000	-1.919740000	-0.922580000
6	3.090754000	-3.106941000	-1.589974000
6	4.432221000	-3.386852000	-1.787743000
6	3.475592000	0.160560000	0.225478000
6	2.145436000	0.565645000	0.477094000
6	1.102099000	-0.285674000	0.006461000
6	1.387754000	-1.486083000	-0.658998000
7	5.086751000	-1.349739000	-0.690774000
6	4.320558000	2.061123000	1.305047000

6	3.021871000	2.514865000	1.583585000
6	1.929373000	1.771875000	1.169826000
6	-0.822366000	-1.162719000	-0.487010000
7	4.550173000	0.910571000	0.639590000
6	-2.258478000	-1.350968000	-0.638437000
6	-2.837661000	-2.513549000	-1.152859000
6	-4.224828000	-2.596171000	-1.278246000
6	-4.734694000	1.885452000	0.551543000
8	-5.331077000	0.687872000	-0.077318000
6	-3.087931000	-0.281295000	-0.256693000
16	-7.324692000	2.267611000	1.587458000
6	-5.813301000	2.950302000	0.640097000
8	-10.284536000	2.431634000	0.433034000
6	-9.935540000	3.316065000	1.556230000
6	-8.494540000	3.770174000	1.382318000
8	-10.877992000	-0.424378000	0.575992000
6	-11.592401000	0.571082000	1.389663000
6	-11.639051000	1.861577000	0.584502000
6	-4.465595000	-0.353607000	-0.392724000
6	-5.052266000	-1.541719000	-0.900762000
8	-6.439668000	-1.555854000	-1.035830000
6	-7.176263000	-2.837021000	-0.821687000
6	-7.592519000	-2.912457000	0.646212000
16	-9.107450000	-4.043323000	0.868977000
6	-10.440706000	-2.798366000	0.307487000
6	-10.639930000	-1.670033000	1.330165000
7	0.154797000	-2.031390000	-0.960164000
44	6.401595000	0.102498000	0.070562000
6	5.847017000	-1.155380000	2.827406000
6	7.787909000	-1.910529000	1.726836000
6	8.066049000	-2.778763000	2.784549000
6	7.209196000	-2.828750000	3.881557000
6	6.082277000	-2.004171000	3.902367000
6	8.623963000	-1.785994000	0.524512000
6	9.810193000	-2.494477000	0.321249000

7	6.679771000	-1.101018000	1.754921000
6	8.872422000	-0.726697000	-1.564605000
6	10.057222000	-1.409728000	-1.811043000
6	10.534461000	-2.307451000	-0.853693000
7	8.160661000	-0.905565000	-0.420999000
6	5.706516000	1.091536000	-2.765469000
6	7.007174000	2.555509000	-1.457345000
6	7.006023000	3.498143000	-2.487606000
6	6.338454000	3.220719000	-3.678135000
6	5.680396000	1.997165000	-3.819213000
6	7.687222000	2.748487000	-0.168886000
6	8.441821000	3.878538000	0.152829000
7	6.354902000	1.356549000	-1.600929000
6	8.146703000	1.813608000	1.943607000
6	8.908044000	2.917825000	2.307603000
6	9.057788000	3.967524000	1.398921000
7	7.541565000	1.721634000	0.730418000
6	5.409888000	-2.491792000	-1.331610000
1	2.335418000	-3.795978000	-1.944787000
1	4.741828000	-4.289900000	-2.295237000
1	5.186665000	2.623373000	1.620084000
1	2.895226000	3.444701000	2.120779000
1	0.915124000	2.093044000	1.366468000
1	-2.234496000	-3.360496000	-1.454451000
1	-4.655050000	-3.499120000	-1.687976000
1	-3.917059000	2.268147000	-0.068999000
1	-4.346790000	1.622352000	1.540435000
1	-2.607188000	0.598011000	0.144514000
1	-5.429194000	3.814858000	1.183007000
1	-6.149568000	3.248704000	-0.354678000
1	-10.061830000	2.796815000	2.513709000
1	-10.593282000	4.194716000	1.548115000
1	-8.341483000	4.172382000	0.378820000
1	-8.233610000	4.514609000	2.134639000
1	-11.073259000	0.737451000	2.342027000

1	-12.611581000	0.225401000	1.601643000
1	-11.996544000	1.653222000	-0.426483000
1	-12.308753000	2.585407000	1.061476000
1	-8.038521000	-2.763820000	-1.485728000
1	-6.574775000	-3.696983000	-1.113114000
1	-7.861092000	-1.918559000	1.006221000
1	-6.805227000	-3.324996000	1.277725000
1	-11.344422000	-3.393147000	0.171943000
1	-10.145931000	-2.388381000	-0.658549000
1	-11.502661000	-1.892947000	1.967852000
1	-9.761119000	-1.544346000	1.972847000
1	-0.011127000	-2.895979000	-1.464186000
1	4.989426000	-0.500314000	2.805353000
1	8.941141000	-3.411198000	2.752501000
1	7.417704000	-3.499661000	4.704256000
1	5.393488000	-2.014349000	4.735786000
1	10.166600000	-3.183376000	1.073085000
1	8.469212000	-0.025026000	-2.278845000
1	10.590910000	-1.236582000	-2.735312000
1	11.454921000	-2.851805000	-1.017851000
1	5.211426000	0.134928000	-2.835134000
1	7.519192000	4.440326000	-2.362218000
1	6.332757000	3.946647000	-4.480345000
1	5.152496000	1.743706000	-4.728138000
1	8.550722000	4.680452000	-0.562657000
1	8.004109000	0.982825000	2.617435000
1	9.371396000	2.948678000	3.284008000
1	9.645112000	4.839475000	1.654310000
1	6.460652000	-2.688048000	-1.483132000
56	-8.633310000	0.774205000	-1.328167000

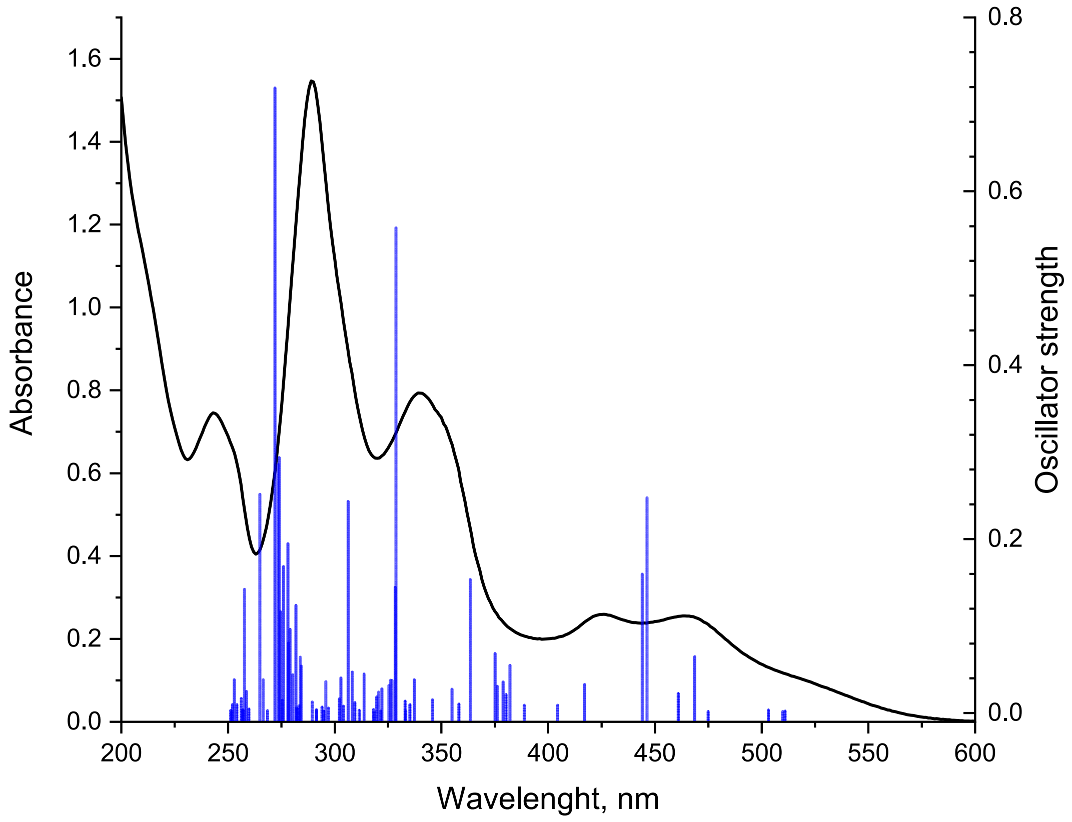

Figure S30. UV–vis absorption (black) spectra of **2** in acetonitrile, along with the oscillator strengths (blue bars) calculated by TD-DFT at the M06/def2-TZVP level of theory

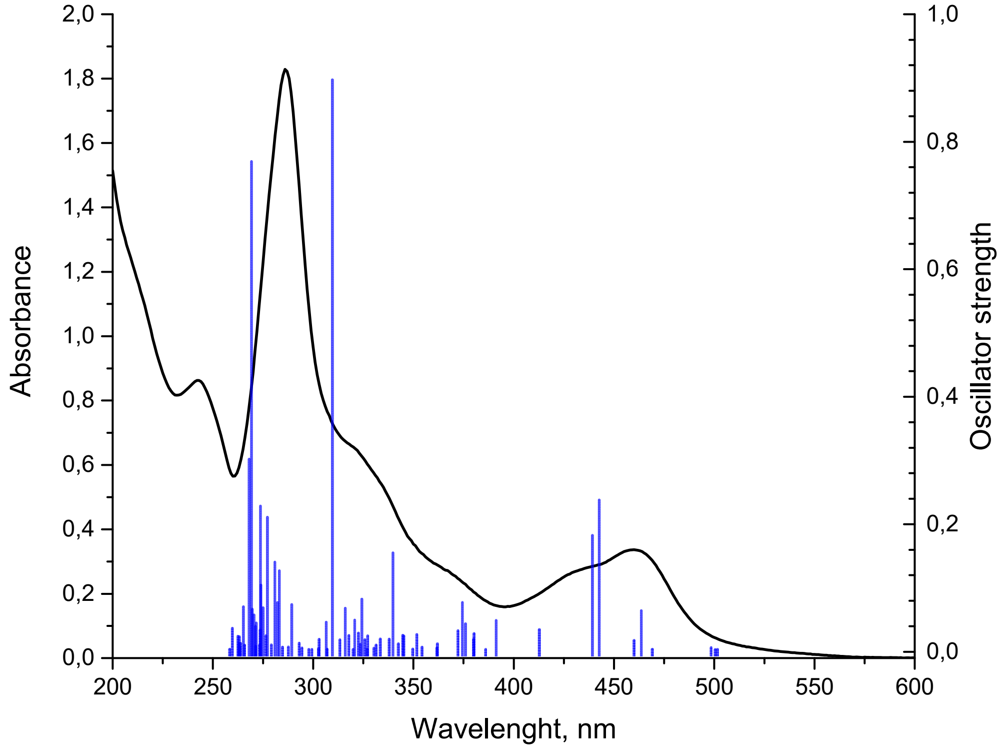

Figure S31. UV–vis absorption (black) spectra of **2●Pb**$^{2+}$ in acetonitrile, along with the oscillator strengths (blue bars) calculated by TD-DFT at the M06/def2-TZVP level of theory

Table S4. Details of the selected transitions in UV-visible spectra of complexes **2** and **2●Pb^{2+}** calculated using TDDFT.

State	ΔE (eV)	Wavelength (nm)	Oscillator Strength	Theoretical Assignment
**2**				
S1	2.423	511.5	0.00082	MLCT + LLCT ($\pi_{ImPh} \rightarrow \pi^*_{bPy}$)
S2	2.429	510.4	0.00031	MLCT ($d_{Ru} \rightarrow \pi^*_{bPy}$) + LLCT ($\pi_{ImPh} \rightarrow \pi^*_{bPy}$)
S3	2.462	503.7	0.00197	MLCT ($d_{Ru} \rightarrow \pi^*_{ImPh}$)
S4	2.608	475.4	0.00035	MLCT
S5	2.643	469.1	0.06344	MLCT
S6	2.687	461.4	0.02100	MLCT ($d_{Ru} \rightarrow \pi^*_{ImPh}$)
S7	2.775	446.7	0.24647	MLCT
S8	2.789	444.4	0.15857	MLCT
S17	3.408	363.8	0.15238	ILCT ($\pi_{ImPh} \rightarrow \pi^*_{ImPh}$)
S25	3.769	328.9	0.55753	IL ($\pi_{ImPh} \rightarrow \pi^*_{ImPh}$) + MLCT ($d_{Ru} \rightarrow \pi^*_{bPy}$)
S40	4.046	306.4	0.24230	IL ($\pi_{ImPh} \rightarrow \pi^*_{ImPh}$)
S56	4.396	282.0	0.12256	IL ($\pi_{bPy} \rightarrow \pi^*_{bPy}$)
S66	4.557	272.1	0.71857	IL ($\pi_{bPy} \rightarrow \pi^*_{bPy}$)
**2●Pb^{2+}**				

S1	2.436	508.8	0.00021	MLCT
S2	2.440	508.0	0.00003	MLCT
S3	2.451	505.7	0.00280	MLCT
S4	2.613	474.4	0.00039	MLCT
S5	2.650	467.8	0.0609	MLCT
S6	2.669	464.5	0.01463	MLCT ($d_{Ru} \rightarrow \pi^*_{ImPh}$) + LLCT ($\pi_{ImPh} \rightarrow \pi^*_{bPy}$)
S7	2.7706	447.5	0.23051	MLCT ($d_{Ru} \rightarrow \pi^*_{ImPh}$) + IL ($\pi_{ImPh} \rightarrow \pi^*_{ImPh}$)
S8	2.799	442.9	0.17473	MLCT ($d_{Ru} \rightarrow \pi^*_{bPy}$)
S24	3.614	343.0	0.14363	ILCT ($\pi_{ImPh} \rightarrow \pi^*_{ImPh}$) + LLCT ($\pi_{ImPh} \rightarrow \pi^*_{bPy}$)
S41	3.952	313.7	0.69712	IL ($\pi_{ImPh} \rightarrow \pi^*_{ImPh}$) + MLCT ($d_{Ru} \rightarrow \pi^*_{bPy}$)
S53	4.349	285.0	0.12566	IL ($\pi_{ImPh} \rightarrow \pi^*_{ImPh}$) + IL ($\pi_{bPy} \rightarrow \pi^*_{bPy}$)
S68	4.569	271.3	0.84855	IL ($\pi_{bPy} \rightarrow \pi^*_{bPy}$)

References:

1. Noble, M.V. and Garrett, A.B., 1944. A Thermodynamic Study of Lead Chloride in Dioxane—Water by Means of Electromotive Force and Solubility Data at 25°; The Acetone-, Ethanol-, Dioxane-, Glycerol—Water—Lead Chloride Systems1a. Journal of the American Chemical Society, 66(2), pp.231-235.

2. Wu, J.Z., Ye, B.H., Wang, L., Ji, L.N., Zhou, J.Y., Li, R.H. and Zhou, Z.Y., 1997. Bis (2, 2′-bipyridine) ruthenium (II) complexes with imidazo [4, 5-f][1, 10]-phenanthroline or 2-phenylimidazo [4, 5-f][1, 10] phenanthroline. Journal of the Chemical Society, Dalton Transactions, (8), pp.1395-1402.
3. Graddon, D.P. and Khoo, C.S., 1988. Thermodynamics of metal-ligand bond formation-XXXVI. Formation of complex zinc and cadmium halides in acetonitrile solution. Polyhedron, 7(21), pp.2129-2133.
4. Gaussian 09, Revision D.01, M. J. Frisch, G. W. Trucks, H. B. Schlegel, G. E. Scuseria, M. A. Robb, J. R. Cheeseman, G. Scalmani, V. Barone, G. A. Petersson, H. Nakatsuji, X. Li, M. Caricato, A. Marenich, J. Bloino, B. G. Janesko, R. Gomperts, B. Mennucci, H. P. Hratchian, J. V. Ortiz, A. F. Izmaylov, J. L. Sonnenberg, D. Williams-Young, F. Ding, F. Lipparini, F. Egidi, J. Goings, B. Peng, A. Petrone, T. Henderson, D. Ranasinghe, V. G. Zakrzewski, J. Gao, N. Rega, G. Zheng, W. Liang, M. Hada, M. Ehara, K. Toyota, R. Fukuda, J. Hasegawa, M. Ishida, T. Nakajima, Y. Honda, O. Kitao, H. Nakai, T. Vreven, K. Throssell, J. A. Montgomery, Jr., J. E. Peralta, F. Ogliaro, M. Bearpark, J. J. Heyd, E. Brothers, K. N. Kudin, V. N. Staroverov, T. Keith, R. Kobayashi, J. Normand, K. Raghavachari, A. Rendell, J. C. Burant, S. S. Iyengar, J. Tomasi, M. Cossi, J. M. Millam, M. Klene, C. Adamo, R. Cammi, J. W. Ochterski, R. L. Martin, K. Morokuma, O. Farkas, J. B. Foresman, and D. J. Fox, Gaussian, Inc., Wallingford CT, 2016.
